# Tubulin Resists Degradation by Cereblon-Recruiting PROTACs

**DOI:** 10.3390/cells9051083

**Published:** 2020-04-27

**Authors:** Ivana Gasic, Brian J. Groendyke, Radosław P. Nowak, J. Christine Yuan, Joann Kalabathula, Eric S. Fischer, Nathanael S. Gray, Timothy J. Mitchison

**Affiliations:** 1 Department of Systems Biology, Blavatnik Institute, Harvard Medical School, Boston, MA 02115, USA; Timothy_Mitchison@hms.harvard.edu; 2 Department of Cancer Biology, Dana-Farber Cancer Institute, Boston, MA 02115, USA; RadoslawP_Nowak@dfci.harvard.edu (R.P.N.); jingting.yuan@som.umaryland.edu (J.C.Y.); Joann_Kalabathula@dfci.harvard.edu (J.K.); Eric_Fischer@dfci.harvard.edu (E.S.F.); Nathanael_Gray@dfci.harvard.edu (N.S.G.); 3 Department of Biological Chemistry and Molecular Pharmacology, Harvard Medical School, Boston, MA 02115, USA

**Keywords:** microtubule, tubulin, PROTAC

## Abstract

Dysregulation of microtubules and tubulin homeostasis has been linked to developmental disorders, neurodegenerative diseases, and cancer. In general, both microtubule-stabilizing and destabilizing agents have been powerful tools for studies of microtubule cytoskeleton and as clinical agents in oncology. However, many cancers develop resistance to these agents, limiting their utility. We sought to address this by developing a different kind of agent: tubulin-targeted small molecule degraders. Degraders (also known as proteolysis-targeting chimeras (PROTACs)) are compounds that recruit endogenous E3 ligases to a target of interest, resulting in the target’s degradation. We developed and examined several series of α- and β-tubulin degraders, based on microtubule-destabilizing agents. Our results indicate, that although previously reported covalent tubulin binders led to tubulin degradation, in our hands, cereblon-recruiting PROTACs were not efficient. In summary, while we consider tubulin degraders to be valuable tools for studying the biology of tubulin homeostasis, it remains to be seen whether the PROTAC strategy can be applied to this target of high clinical relevance.

## 1. Introduction

The microtubule cytoskeleton is a network of filaments composed of α- and β-tubulins—exceptionally abundant proteins that form soluble heterodimers (hereafter αβ-tubulin), which polymerize into dynamic microtubules [1]. The biomechanical properties and functions of the microtubule cytoskeleton critically depend on tubulin abundance, which is regulated through balanced protein synthesis and degradation [2,3,4]. In non-dividing cells, microtubules mediate intracellular transport and organize the cytoplasm [5]. In dividing cells, microtubules build the mitotic spindle—a complex and dynamic machinery that powers division of the genetic material [6]. Due to such a broad spectrum of important functions, cells are extraordinarily sensitive to defects in microtubule and tubulin homeostasis, which cause devastating phenotypes, such as developmental disorders, neurodegenerative diseases, and cancer [7]. Microtubule and tubulin homeostasis can be impaired by a wide range of physiological and toxic inputs, including natural and synthetic molecules that bind to and inhibit polymerization or depolymerization of tubulin [8,9].

Numerous compounds have been identified to bind tubulin, with the majority belonging to one of five structurally distinct classes: the auristatins, colchicines, combretastatins, taxanes, and vinca alkaloids [9]. These molecules act as reversible inhibitors, without impacting tubulin’s native conformation or stability. The auristatin and vinblastine families of ligands destabilize microtubules by binding at the interface of αβ-tubulin [10,11,12], whereas colchicine and combretastatin ligands destabilize microtubules by binding to β-tubulin [13,14]. In contrast, paclitaxel (taxol) and related analogues also bind to β-tubulin, yet stabilize microtubules [15,16]. Both microtubule-stabilizing and destabilizing agents have been powerful research tools for studies of microtubules and tubulins, and widely used chemotherapeutics for cancer treatment [17]. However, the commonly used clinical microtubule-targeting agents, such as paclitaxel and vinblastine, are susceptible to drug resistance [18]. Moreover, despite the established role of microtubule dysregulation and tubulin aggregation in neurodegenerative disease, there are currently no effective tubulin-targeting agents that are useful in this context [19]. Taken together, these existing limitations emphasize an urgent need to develop tubulin-targeting agents with novel mechanisms of action (MOA).

One such class of targeting agents with a unique MOA are small molecules that mediate targeted protein degradation, an emerging strategy in drug development. Degrader molecules recognize both a protein target of interest and an E3 ubiquitin ligase, thus bringing the target in close proximity to an enzyme that earmarks it for degradation by the ubiquitin-proteasome system (UPS) [20]. The therapeutic potential of targeted protein degradation is highly promising for a number of reasons: (1) in contrast to the standard model of inhibition, high affinity ligands and continuous target occupancy may not be required due to the catalytic mode of action of degraders, therefore enabling development of degraders for targets considered to be “undruggable” (*aka* “unligandable”); (2) the protein of interest is degraded, ablating not only the pathology associated with enzymatic activity of the target, but also any scaffolding functions or protein–protein interactions; and (3) degrader molecules operate at sub-stoichiometric concentrations, which may enable lower dosing and improved side-effect profiles. Given these advantages, development of degrader molecules has progressed rapidly, and two PROTAC degraders, the most common degrader molecules, have recently entered into clinical trials (ARV-110 targeting the androgen receptor [21] and ARV-471 targeting the estrogen receptor [22]). Pre-clinical development of PROTACs has also been accelerating, and they have been successfully used to degrade a wide range of targets, including BET-family proteins [23,24], androgen and estrogen receptors [25,26], hepatitis C virus (HCV) protease [27], histone deacetylase 6 (HDAC6) [28], aberrant tau [29], and numerous protein kinases [30,31,32,33].

Small molecules that result in degradation of α- and β-tubulin are not without a precedent in the current literature [34,35,36]. It has been known for some time that molecules that covalently modify Cys-239 on β-tubulin lead to loss of both α- and β-tubulin, and that this process is mediated by the proteasome [35,36]. Thus, given the stated challenges surrounding existing microtubule-targeting modalities, and the evidence that small molecule-mediated tubulin degradation is achievable, we set out to develop tubulin-targeting PROTACs. We synthesized and tested a panel of PROTACs based on monomethyl auristatin E (MMAE, vedotin) or combretastatin A-4 (CA4) as tubulin-ligands, and cereblon (CRBN) E3-ligase-binding small molecules, connected via linkers of various lengths and chemical properties. The reported covalent compounds, T007-1 [35] and T138067 [36], were used as benchmark reagents for establishing tubulin degradability. Our results show that tubulin remains resistant to degradation using CRBN-recruiting PROTAC degraders, suggesting potential limitations of the PROTAC technology when targeting highly abundant proteins with complex homeostasis.

## 2. Materials and Methods

### 2.1. Cell Culture and Drug Treatments 

The human telomerase reverse transcriptase immortalized retinal pigment epithelium 1 (hTert-RPE1) cell line used in this study was grown at 37 °C with 5% CO_2_ in a humidified incubator. Cells were grown in Dulbecco’s modified medium (nutrient mixture F12, DMEM/F12) supplemented with 10% fetal bovine serum, and 1% (*v*:*v*) penicillin/streptomycin (pen/strep). The Flp293T-based BRD4_BD2_-GFP mCherry reporter cell line was grown in DMEM supplemented with 10% FBS. The synthesized small molecules and the parental microtubule poisons were dissolved in DMSO; 0.01% DMSO was used as vehicle control. All the compounds were administered at indicated concentrations for five hours prior to assay for two reasons: (1) our time-course experiment (data not shown) indicated that the reference compounds, T007-1 and T138067, efficiently degrade tubulin in that timeframe and (2) to avoid accumulation of cells arrested in mitosis that could bias the analysis of tubulin abundances across the samples.

### 2.2. Western Blotting and Tubulin Partitioning

Whole-cell extracts for immunoblot analysis were prepared by cell lysis in 2× SDS sample buffer composed of 50 mM Tris-HCl pH 6.8, 2% SDS, 10% glycerol, 143 mM β-mercaptoethanol, 12.5 mM EDTA, and 0.02% bromophenol blue in water. Samples were denatured at 100 °C for 10 min and then subjected to SDS-PAGE gel electrophoresis on 1.5 mm NuPAGE Novex 4–12% Bis-Tris Protein Gels (Thermo Fisher Scientific, Waltham, MA, USA), using the Mini Trans-Blot Cell system (BioRad, Hercules, CA, USA).

For tubulin partitioning into soluble and polymerized fractions, cells were grown in 12-well dishes at 70–80% confluence. Cells were gently washed with PBS pre-warmed to 37 °C. Soluble tubulin was extracted by incubating cells in 300 µL of tubulin extraction buffer containing 60 mM PIPES (pH 6.8), 25 mM HEPES (pH 7.2), 10 mM EGTA (pH 7–8), 2 mM MgCl_2_, 0.5% TritonX-100, 10 µM Paclitaxel (Sigma Aldrich, St. Louis, MO USA), and a protease inhibitor tablet (Roche, Branchburg, NJ, USA) for 2 min at room temperature. The extraction buffer with soluble protein was collected and mixed with 100 µL 4× SDS sample buffer. The remaining material containing polymerized tubulin was then collected by adding 400 µL 1× SDS sample buffer and scraping the dish. All samples were denatured at 100 °C for 10 min. Equal aliquots of samples were further concentrated two-fold by evaporation at 100 °C for 3–5 min immediately prior to SDS-PAGE gel electrophoresis.

The Precision Plus Protein Dual Color Standard ladder was used for all immunoblots (BioRad, USA). Primary antibodies were diluted: 1:5000 beta-actin (Cell Signaling Technologies, Danvers, MA, USA, #4970), 1:15000 GAPDH (Cell Signaling Technologies, #2118), 1:10000 alpha-tubulin (DM1alpha, Millipore, Burlington, MA, USA, #05-829), and 1:5000 beta-tubulin (Cell Signaling Technologies, # 2128). Secondary antibodies were diluted: 1:15000 goat anti-mouse DyLight 680 conjugated (Thermo Fisher Scientific, #35518), 1:15000 goat anti-rabbit DyLight 800 conjugated (Thermo Fisher Scientific, #35571). The Odyssey Infra-Red Imaging System (LI-COR, Lincoln, NE, USA) equipped with the Image Studio software was used to image and analyze immunoblots. Data plotting and statistical analysis were carried out in Microsoft Excel (Redmond, WA, USA) and R (Vienna, Austria).

### 2.3. General Chemistry Methods

Full experimental procedures and characterization of new compounds are provided in the Supplementary Material. Analytical-grade solvents and commercially available reagents were purchased from commercial sources and used directly without further purification unless otherwise stated. Monomethyl auristatin E (MMAE) was purchased from Biorbyt (St. Louis, MO, USA) and MedChemExpress (Monmouth Junction, NJ, USA). Experiments were conducted under ambient conditions unless otherwise stated. Thin-layer chromatography (TLC) was carried out on Merck 60 F_254_ precoated, glass silica plates which were visualized with either ultraviolet light or stained with KMnO_4_. ^1^H-NMR, ^13^C-NMR, and ^19^F-NMR spectra were recorded at room temperature using a Bruker 500 (^1^H-NMR at 500 MHz, ^13^C-NMR at 125 MHz, and ^19^F-NMR at 471 MHz). Chemical shifts are reported in ppm with reference to solvent signals [^1^H-NMR: CDCl_3_ (7.26 ppm), DMSO-*d*_6_ (2.50 ppm); ^13^C-NMR: CDCl_3_ (77.16 ppm), DMSO-*d*_6_ (39.52 ppm)]. Signal patterns are indicated as s, singlet; br s, broad singlet; d, doublet; t, triplet, q, quartet; p, pentet; and m, multiplet. Mass spectrometry (MS) analysis was conducted on a Waters Acquity UPLC-MS system using electrospray ionization (ESI) and reporting the molecular ion [M + H]^+^, [M + Na]^+^ or a suitable fragment ion. Flash chromatography purification was conducted using an ISCO CombiFlash RF+ with RediSep Rf silica cartridges. Preparative reverse-phase HPLC purification was conducted using a Waters model 2545 pump and 2489 UV/Vis detector using SunFire Prep C18 5 μm columns (18 × 100 mm, 20 mL/min flow rate; 30 × 250 mm, 40 mL/min flow rate), and a gradient solvent system of water (0.035% TFA)/methanol (0.035% TFA) or water (0.035% TFA)/acetonitrile (0.035% TFA).

### 2.4. Docking and Linker Length Analysis

Structural illustration of our tubulin degradation strategy was obtained using Illustrate [37]. All protein docking was carried out using Rosetta 3.8 provided through SBGrid [38]. Crystallization of tubulin is challenging without the presence of tubulin-binding proteins, explaining the presence of such proteins in most published crystal structures of tubulin. Given that stathmin is one of the main binding partners of soluble tubulin in cells, we chose to perform the docking analysis with a tubulin/stathmin crystal structure. Input models were downloaded from the PDB (CRBN-bound lenalidomide PDB ID 5fqd, chain B; tubulin PDB ID 4x1i, chain B and C, ligand removed). The ligand parameters for lenalidomide were generated using OpenEye Omega (OpenEye Scientific) and parameter files generated in Rosetta, using the command ‘molfile_to_params.py’. The PDB structure coordinates of the input files were merged into a single file and prepared for docking using the Rosetta program ‘docking_prepack_protocol’ program. The first round of global docking was performed by running the Rosetta program ‘docking_protocol’ 80 times (for a total of 16,000 poses) with the following command line options: *partners A_B -dock_pert 5 25 -randomize2 -ex1 -ex2aro -nstruct 200 -beta,* providing the combined PDB, lenalidomide-specific parameter files, and database file as input.

To analyze the range of possible low-energy binding modes for tubulin, the top 200 lowest I_sc scoring docking decoys were selected. An X-ray crystal structure model of tubulin bound auristatin-0101 (PDB ID 4x1i) or combretastatin A-4 (PDB ID 5lyj, chain D) was superimposed to each of the docked poses of tubulin in Pymol using the ‘align’ command (The PyMOL Molecular Graphics System, Version 1.8.6.0 Schrödinger, LLC). The shortest pairwise distance between selected atoms for potential linker attachment on lenalidomide and auristatin-0101 was calculated in Pymol for each of the top 200 poses (Euclidean distance). The histogram was generated in GraphPad Prism 7 with the Column Analysis–Frequency Distribution tool. Data analysis and statistics for all steps were performed using GraphPad Prism 7.

## 3. Results

### 3.1. Cereblon-Based PROTACs with Auristatin Scaffold

**Figure 1:**
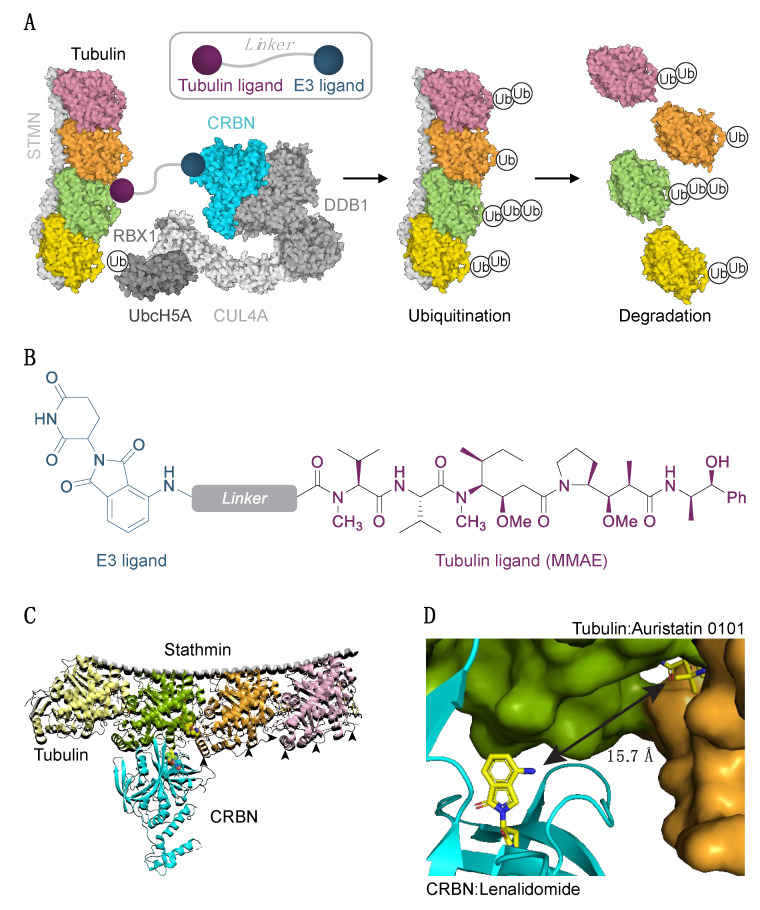
Experimental strategy to degrade tubulin using the PROTAC technology. (**A**) Schematic diagram of protein degradation strategy. Recruitment of the E3 ligase to tubulin heterodimer via the tubulin/E3 ligand molecule would cause tubulin ubiquitination (Ub) and subsequent proteasome-mediated degradation. Structure of two tubulin heterodimers (pink, orange, green, yellow) bound to stathmin (STMN, PDB ID 4x1i) and the CRL4^CRBN^ E3 ligase complex is shown (RBX1/CUL4/DDB1, PDB ID 4a0K; CRBN PDB ID 5fqd; UbcH5A, PDB ID 2c4p). (**B**) Structure of a chimeric compound. (**C**) Docking pose of CRBN and tubulin. Arrowheads indicate some of the exposed lysine residues in tubulins. (**D**) The shortest pairwise distance between lenalidomide and auristatin-0101 from pose in (**C**) is permissive of the degrader approach.

Our initial PROTAC design was based on the use of microtubule-destabilizing agent auristatin (or its derivatives) as the tubulin recruiting ligand. We excluded other classes of tubulin ligands, such as taxanes and vinca alkaloids, from our first round of design due to the synthetic difficulties inherently associated with derivatizing complex natural products. Pomalidomide would serve as the cullin ring E3 ligase recruiter arm (CRL4^CRBN^; E3 ubiquitin ligase, Figure 1A,B), linked to the tubulin ligand via a flexible linker. Attachment of the linker at a solvent-exposed site on the molecule allows the PROTAC to retain binding affinity for the protein of interest. Rapid optimization of the linker remains a challenge in PROTAC design, due to a large chemical space and potential variability. Additionally, the conformation adopted by the linker has been shown to play an important role in productive ternary complex formation between the target, PROTAC molecule, and the E3 ligase [24]. The linker choice, therefore, requires careful consideration. To facilitate the PROTAC design process and jump-start our chemistry efforts, we used a previously developed molecular docking strategy [24,39] to predict the optimal linker length. We subjected tubulin (PDB ID 4x1i, chain B and C) and pomalidomide-bound CRBN (PDB ID 5fqd, chain B) to protein–protein docking in the Rosetta framework generating 16,000 poses, from which we filtered the top 200 by the interface score (Figure 1C,D, Supplementary Figure S1A–D). In order to calculate linker length, we aligned tubulin-bound auristatin 0101 (dolastatin 10 analog) to its docked pose and calculated pairwise distances between selected atoms on tubulin and lenalidomide (Supplementary Figure S1A–D). These simulations allowed us to predict two clusters in optimal linker lengths: (1) short 7–10 Å distance corresponding to the exit vector coming out of the phenyl ring of auristatin 0101, requiring a linker of 6–8 atoms, and (2) long >15 Å distance corresponding to primary amine nitrogen, requiring a linker of 11 or more atoms (Figure 1D, Supplementary Figure S1A–D).

**Figure 2:**
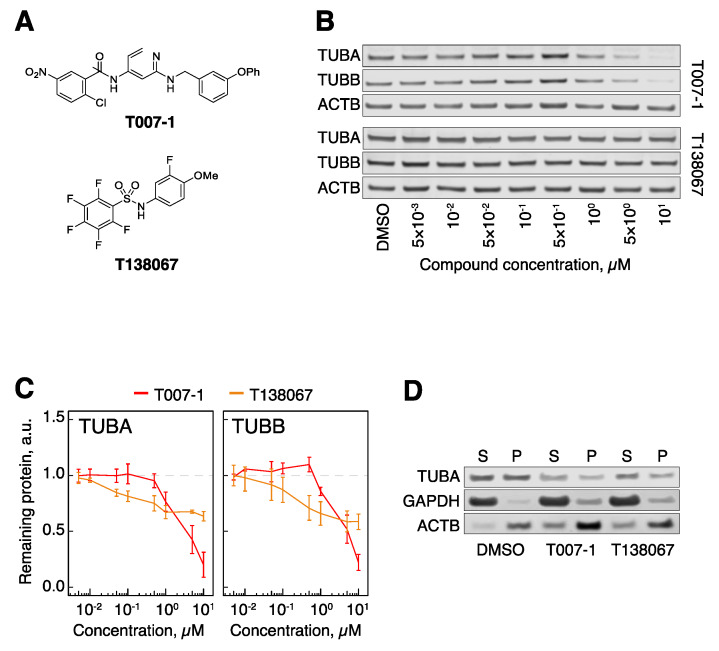
Covalent binders target tubulin for degradation. (**A**) Structural formulae of T007-1 and T138067. (**B**) TUBA and TUBB protein levels in hTert-RPE1 cells upon treatment with T007-1 and T138067. (**C**) Quantification of tubulin protein levels across the indicated conditions. All data are normalized to reference protein (ACTB) and tubulin protein levels in DMSO-treated cells. Lines represent the average of three biological replicates. Error bars represent standard error of the mean (SEM). (**D**) Biochemical partitioning of tubulin into soluble (S) and polymerized (P) forms across the indicated conditions. Note that both the S and the P fraction of tubulin decrease upon treatment with 1 μM T007-1 and T138067 for 5 h.

As positive controls, we prepared two of the literature molecules, T007-1 and a related T138067 (Figure 2A) [35]. We modified the originally reported synthesis of T007-1, instead using 2-chloro-5-nitrobenzoyl chloride, as the reported dicyclohexyl carbodiimide (DCC) coupling with 2-chloro-5-nitrobenzoic acid led to inseparable mixtures of mono- and diacylation (See Supplementary Material for full details). We treated human retinal pigment epithelium 1 (hTert-RPE1) cells, a widely used cell model, with T007-1 and T138067 degraders at a range of concentrations from 5 nM to 10 μM. Prolonged compound treatment was expected to cause activation of the spindle assembly checkpoint and an arrest in the mitotic stage of the cell cycle [40], which could have an effect on tubulin levels independently of tubulin degradation. To exclude this possibility, we used contact-inhibited quiescent cells (hTert-RPE1 cells stop proliferating at high growth density) [3]. In addition, to minimize the probability that some cells escape the cell cycle arrest and continue to proliferate, we opted for a short 5 h treatment of cells with the compounds, which was previously reported to be sufficient to degrade tubulin [35]. Western blot analysis of α-tubulin (TUBA) and β-tubulin (TUBB) revealed that both compounds induced a decrease in tubulin levels, with T007-1 being significantly more potent in our hands (Figure 2B,C). Our follow-up analysis of the effects of these compounds showed that T007-1-induced degradation of TUBA and TUBB led to decreased levels of both soluble (S) and polymerized (P) fractions of tubulin (Figure 2D). Therefore, as previously reported, this series of compounds led to degradation of tubulin and a global decrease of both soluble and polymerized tubulin levels. These results served as a benchmark for our studies of PROTAC molecules.

**Figure 3:**
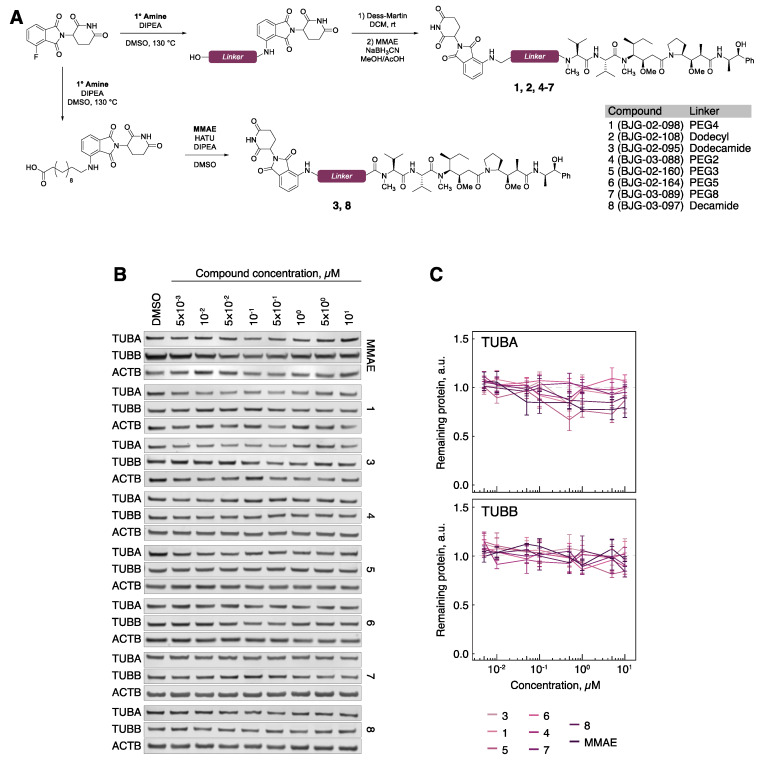
Tubulin evades MMAE-CRBN targeted degradation. (**A**) Chemical synthesis of MMAE-CRBN compounds with varying linkers. (**B**) TUBA and TUBB protein levels in hTert-RPE1 cells upon treatment with the indicated compounds. (**C**) Quantification of tubulin protein levels across the indicated conditions. All data are normalized to ACTB, then to tubulin protein levels in DMSO-treated cells. Lines represent the average of three biological replicates. Error bars represent SEM.

We chose monomethyl auristatin E (MMAE) as the tubulin ligand of choice due to synthetic tractability for functionalization through the secondary amine, corresponding to cluster 2 in the docking simulations (Supplementary Figure S1B). Three candidate degraders were prepared accordingly, containing a PEG4 (1, BJG-02-098), dodecyl (2, BJG-02-108), or dodecamide (3, BJG-02-095) linker (Figure 3A, Supplementary Figure S2). Compounds containing a tertiary amine linkage were synthesized by reductive amination of MMAE with the respective linker-aldehyde, which was obtained by oxidation with Dess–Martin periodinane (DMP) and used without purification. Compounds containing an amide linkage were prepared by hexafluorophosphate azabenzotriazole tetramethyl uronium (HATU) coupling of MMAE with the requisite carboxylic acid.

Compared to typical small molecule inhibitors, such as T007-1, PROTACs have higher molecular weight, which may decrease their cell permeability. In order to gauge the effects of linker composition on cellular availability, compounds 1–3 (Figure 3A) were evaluated by a previously developed cellular CRBN engagement assay [24]. We observed that 1 is cell-permeable, with an IC_50_ of 342 nM (Supplementary Figure S3). In contrast, 2 showed no CRBN engagement, and 3 showed very weak CRBN engagement, with an IC_50_ of ~10 μM (Supplementary Figure S3). Treatment of hTert-RPE1 cells with 1 and 3 (1 μM) for 5 h resulted in microtubule destabilization, as evidenced by an increased fraction of soluble tubulin and a reduced fraction of polymerized tubulin (Supplementary Figure S4A), confirming that these compounds retained the microtubule-destabilizing properties of the parent inhibitor, MMAE. With evidence that 1 and 3 successfully bound both of their desired targets, we proceeded to test for degradation of tubulin. However, upon treatment of hTert-RPE1 cells with 1 or 3 ranging from 5 nM to 10 μM for 5 h and subsequent Western blotting, no degradation of either TUBA or TUBB was observed (Figure 3B,C).

Because 1 and 3 retained the cellular activity of both the E3 ligase recruiter and the tubulin binder, we surmised that the linker length may be suboptimal. To explore linker optimization further, we prepared a suite of molecules containing both longer (PEG5, PEG8) and shorter (PEG2, PEG3, decamide) linkers (Figure 3A and Supplementary Figure S2). Despite efficient CRBN-binding and microtubule-destabilizing activity (Supplementary Figures S3 and S4B), these molecules also failed to degrade tubulin (Figure 3B,C).

### 3.2. Cereblon-Based PROTACs with the Combretastatin A-4 Scaffold

**Figure 4:**
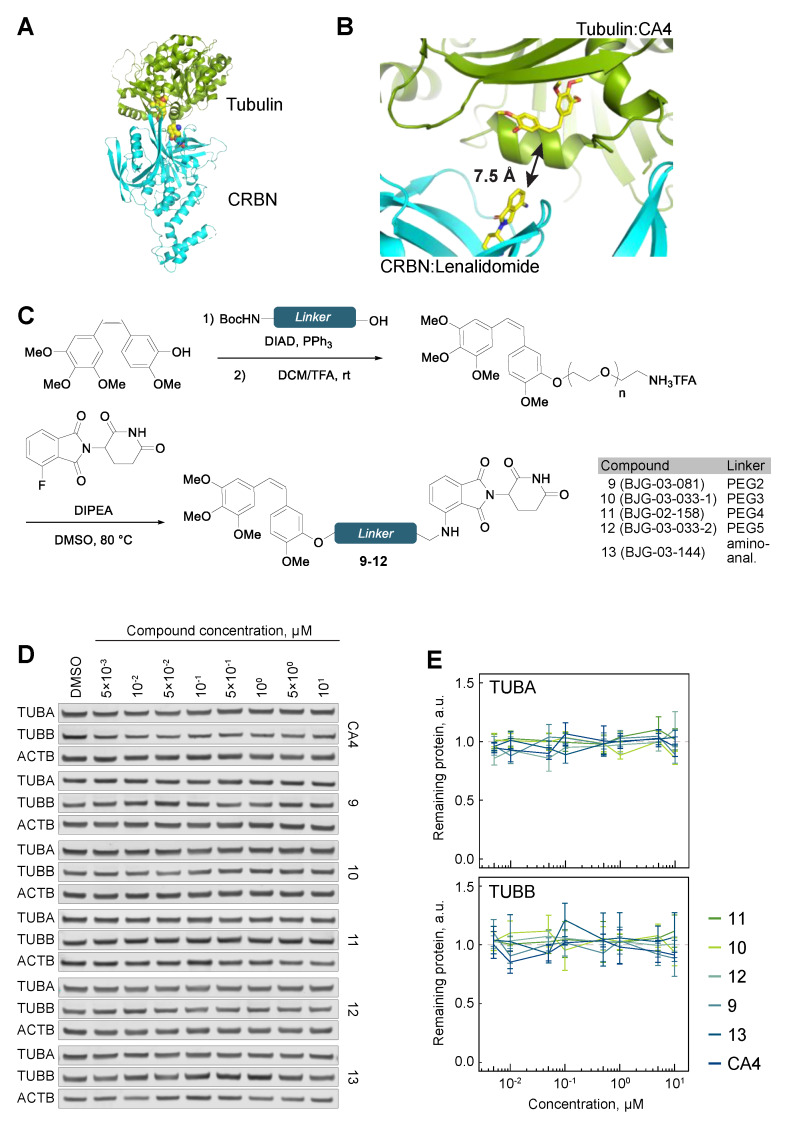
Tubulin evades CA4-CRBN targeted degradation. (**A**) The shortest linker docking pose of CRBN-bound lenalidomide (PDB ID 5fqd) and tubulin-bound CA4 (PDB ID 5lyj). (**B**) Corresponding shortest pairwise distance between lenalidomide and CA4 is permissive of a degrader approach. (**C**) Chemical synthesis of CA4-CRBN a degrader compounds with varying linkers. (**D**) TUBA and TUBB protein levels in hTert-RPE1 cells upon treatment with the indicated compounds. (**E**) Quantification of tubulin protein levels across the indicated conditions. All data are normalized to ACTB, then to tubulin protein levels in DMSO-treated cells. Lines represent average of three biological replicates. Error bars represent SEM.

This inability to degrade tubulin by PROTACs may be the result of an unstable ternary complex, or a slow rate of ubiquitylation. We reasoned that either of these potential roadblocks could be overcome by targeting a different binding site on the tubulin subunit, and therefore we pursued a tubulin PROTAC design using another microtubule destabilizer, combretastatin A-4 (CA4). In particular, we chose CA4 because of its ease of synthetic access and the ability for modular derivatization through 3′-hydroxyl. Unlike the auristatin scaffold that binds at the interface of the TUBA and TUBB dimer, CA4 binds only TUBB. Docking analysis confirmed a linker distance as short as 7.5 Å would be suitable for lenalidomide and CA4 PROTACs (Figure 4A,B and Supplementary Figure S1E,F). A series of compounds 9–12, containing linker lengths spanning PEG2 to PEG5, was synthesized from CA4 via Mitsunobu coupling with a Boc-protected amino alcohol, followed by deprotection and S_N_Ar reaction with 3-fluorothalidomide (Figure 4C and Supplementary Figure S5). Partial isomerization of the labile *Z*-alkene occurred under the reaction conditions, but the alkene isomers of the final compounds were separable by reverse-phase preparative HPLC. Compounds **9–12** were cell permeable, as judged by the cellular CRBN engagement assay (IC_50_ ~600 to 1500 nM, Supplementary Figure S6). However, partitioning of tubulin into soluble and polymerized fractions revealed that all the compounds failed to significantly destabilize the microtubule cytoskeleton as seen from the comparison of the S and P fractions across the indicated conditions, suggesting that they lost the activity of the parental CA4 (Supplementary Figure S7). Analyses of total cellular extracts revealed no sign of tubulin degradation upon treatment with 9–12 (Figure 4D,E).

Despite the extensive structure–activity relationship (SAR) investigations that have been conducted on CA4 [41,42,43], relatively few examples contain long alkyl substituents at the 3′-hydroxyl position, although shorter substitutions at this position were found to reduce potency [44]. Given that the hydroxyl group forms a hydrogen bond with tubulin (Asn258, PDB ID 5lyj), we hypothesized that an amino-analogue of CA4 may display increased potency by retaining the ability to hydrogen bond. To this end, 13, a 3′-amino analogue of 11, was synthesized (Figure 4C and Supplementary Figure S5). While this compound showed efficient binding to CRBN (Supplementary Figure S6), it displayed no microtubule-destabilizing activity, as seen by unperturbed partitioning of tubulin into soluble and polymerized forms (Supplementary Figure S7), and was unsuccessful in degrading tubulin (Figure 4D,E).

## 4. Discussion

Tubulin-targeting agents have been used successfully as chemotherapeutic drugs in cancer treatment for decades [9]. More recently, dysregulation of tubulin homeostasis has been implicated in areas beyond oncology, most notably neurodegenerative diseases [19]. Unfortunately, currently available agents show no efficacy in these systems, suggesting that development of new tubulin-targeting agents with fundamentally novel mechanisms of actions may be needed to move into additional therapeutic indications. Degrader molecules represent one such modality because, unlike the majority of bioactive small molecules, they induce proteasomal degradation of the target instead of inhibiting its function. Tubulin, as a degradation target, is an interesting case, given its complex homeostasis. First, monomeric TUBA and TUBB are unstable and only exist during their biogenesis in cells [45], making the TUBA–TUBB heterodimer (αβ-tubulin) a basic form of tubulin in vivo. Second, αβ-tubulins partition and continually exchange between soluble and polymerized (microtubule) forms—a behavior termed microtubule dynamic instability [46]. In the majority of cell types, most αβ-tubulins are incorporated into microtubules at any given time. However, protein degradation machineries presumably have access to soluble but not polymerized αβ-tubulins [2,47]. Finally, levels of αβ-tubulins are tightly controlled at the mRNA level, where stability of tubulin mRNA is negatively regulated by soluble tubulin [2,3,4,48]. This negative feedback is active in instances of excess soluble αβ-tubulin, rapidly decreasing the supply of newly synthesized tubulins. Together, these complex aspects of tubulin biology raise a question of whether and how targeted degradation of tubulin would be achievable. Interestingly, compounds that covalently react with different cysteines on TUBB, most notably Cys-239, have been known to reduce both TUBB and TUBA levels via proteasomal degradation [35]. Our results using examples from one series of these compounds, exemplified by the recently reported T007-1 [35], confirmed previous observations and suggested that targeted degradation of tubulin via PROTAC-type molecules may be feasible.

To test this hypothesis, we designed and developed PROTAC molecules that recruit the E3 ubiquitin ligase CRBN and deploy various tubulin-recruiting moieties. Specifically, we used an auristatin-based scaffold (MMAE), which binds to the TUBA–TUBB interface, as well as the combretastatin A-4 (CA4) scaffold that engages only TUBB as tubulin recruiting arms of our PROTACs. Based on the results obtained using this initial collection of tubulin-targeted PROTACs, we conclude that tubulin protein is recalcitrant to degradation mediated by CRBN-recruiting PROTACs.

Negative results on degradation were obtained over a broad range of rationally designed PROTACs and suggest that this is not a simple technical artifact of failed binding. This is further supported by the excellent CRBN engagement in cells and cellular tubulin binding we observed using MMAE–CRBN degraders. We speculate that for MMAE–CRBN degraders, failure to degrade tubulin may be due to inability of these compounds to mediate formation of a productive tubulin–PROTAC–CRBN ternary complex. This is likely due to kinetic considerations, as our initial docking studies of dolastatin 10-bound tubulin (auristatin analog) and pomalidomide-bound CRBN suggested that formation of a ternary complex could be thermodynamically allowable, without major steric or electrostatic clashes. It is possible that the ternary complex formation is kinetically disfavored or that tubulin’s surface lysines may be inaccessible for successful ubiquitin transfer. Further investigations are required to determine whether the observed resistance to PROTAC-mediated degradation is universal, or if molecules that target other E3 ligases, such as VHL, cIAP, or RNF114, can successfully degrade tubulin.

In contrast to the MMAE-CRBN degraders, the CA4-based degraders bearing a linker attachment at the 3′ hydroxyl position lost the tubulin-destabilizing ability of the parent inhibitor. This result is somewhat surprising, as analysis of the crystal structure of tubulin-bound CA4 suggested that this site was modifiable. The lack of binding affinity is most likely due to a loss of hydrogen bonds from the hydroxyl group, but it is also possible that the longer alkyl substituent disrupts the ability of the CA4 core to fit into the binding site. Inspection of the crystal structure suggests that the 2′ C–H and the 4′ methoxy sites are viable for linker attachment and may provide degrader analogs with improved tubulin affinity. Alternatively, it is conceivable that CA4-based degraders bind to tubulin and microtubules, but that the recruitment of CRBN disrupts the ability of tubulin to dissociate from microtubules. Further studies are necessary to determine if different CA4 or other tubulin inhibitor derivatives may target tubulin for degradation. Clinically successful vinca alkaloids or taxanes would be ideal parental molecule candidates for the PROTAC approach. However, both of these classes of molecules lack reactive groups that would allow straightforward derivatization. An added complication with taxanes is that they make tubulins inaccessible for proteolysis by promoting their polymerization [46].

Although disappointing, these results are not unique to tubulin. Recently published results on exploring the degradation of KRAS^G12C^, which also did not yield a functional PROTAC [49], suggest that PROTAC technology may have some limitations. Although vastly different, tubulin and KRAS^G12C^ are both protein systems with complex homeostasis that is incompletely understood. In both cases, these proteins spend significant time in association with a membrane (in the case of KRAS^G12C^), or microtubules and heterodimer (in the case of tubulin). Under these circumstances, it is unclear whether productive formation of a ternary complex, which is required for efficient polyubiquitination of the target and subsequent degradation, is achievable. One reason why compounds such as T007-1 are speculated to work is that they cause destabilization of TUBB, resulting in dissociation of the heterodimer, thus leading to both TUBB and TUBA being recognized by chaperone or other systems that promote clearance of misfolded proteins. This is markedly different from how PROTACs would work. Current efforts by multiple groups are directed at elucidating the “rules” that govern whether a given target can be degraded by a given E3 ligase. In the meantime, our work emphasizes that while the PROTAC concept is one of the most exciting developments in modern pharmacology, it is not universally applicable.

## Supplementary Materials

The following are available online at https://www.mdpi.com/2073-4409/9/5/1083/s1, Supplementary Materials and Methods, Supplementary Figure S1: Analysis of linker length and score of top 200 docked poses for CRBN and tubulin, Supplementary Figure S2: MMAE-CRBN compounds, Supplementary Figure S3: MMAE-CRBN compounds engage CRBN in cells, Supplementary Figure S4: MMAE-CRBN compounds destabilize microtubule cytoskeleton, Supplementary Figure S5: CA4-CRBN compounds, Supplementary Figure S6: 6. CA4-CRBN compounds engage CRBN in cells, Figure S7: CA4-CRBN compounds lack microtubule-destabilizing activity.

## Abbreviations

a.u.	arbitrary units
CA4	combretastatin A-4
CRBN	cereblon
CUL4A	cullin 4A
DCC	dicyclohexyl carbodiimide
DDB1	damage specific DNA binding protein
DMP	Dess-Martin periodinane
DMSO	dimethyl sulfoxide
HATU	hexafluorophosphate azabenzotriazole tetramethyl uronium
hTert	human telomerase reverse transcriptase
IC	inhibitory concentration
KRAS	Ki-ras2 Kirsten rat sarcoma viral oncogene homolog
MMAE	monomethyl auristatin E
MOA	mechanism of action
P	polymerized
PDB	protein data bank
PROTAC	proteolysis-targeting chimera
RBX1	ring box 1
RPE1	retinal pigment epithelium 1
S	soluble
SAR	structure-activity relationship
SEM	standard error of the mean
STMN	stathmin
TFA	trifluoroacetic acid
TLC	thin-layer chromatography
TUBA	α-tubulin
TUBB	β-tubulin
UbcH5A	ubiquitin-conjugated enzyme E2D1
Ub	ubiquitin
UPS	ubiquitin-proteasome system
