# Supplementary material for: Tubulin Resists Degradation by Cereblon-Recruiting PROTACs
Source: Cells. 2020 Apr 27;9(5):1083. doi: 10.3390/cells9051083 (PMC7290497; doi:10.3390/cells9051083)
Supplement: Supplementary file 1 [file cells-09-01083-s001.pdf]

Supplementary Data, Materials and Methods

Tubulin Resists Degradation by Cereblon-Recruiting PROTACs

Ivana Gasic [1,†,*], Brian J. Groendyke [2,3,†,*], Radosław P. Nowak [2,3], J. Christine Yuan [2,3], Joann Kalabathula [2,3], Eric S. Fischer [2,3], Nathanael S. Gray [2,3] and Timothy J. Mitchison [1]

[1] Department of Systems Biology, Blavatnik Institute, Harvard Medical School, Boston, Massachusetts 02115, USA; Timothy_Mitchison@hms.harvard.edu (T.J.M.)
[2] Department of Cancer Biology, Dana-Farber Cancer Institute, Boston, Massachusetts 02115, USA; RadoslawP_Nowak@dfci.harvard.edu (R.P.N.); jingting.yuan@som.umaryland.edu (J.C.Y.); Joann_Kalabathula@dfci.harvard.edu (J.K.); Eric_Fischer@dfci.harvard.edu (E.S.F); Nathanael_Gray@dfci.harvard.edu (N.S.G.)
[3] Department of Biological Chemistry and Molecular Pharmacology, Harvard Medical School, Boston, Massachusetts 02115, USA
* Correspondence: Ivana_Gasic@hms.harvard.edu (I.G.); Groendyke@dfci.harvard.edu (B.J.G.)
† Equal contribution.

Table of contents:

1. **Supplementary Figures and Figure Legends**
2. **General methods**
3. **Competitive displacement assay for cellular CRBN engagement**
4. **Experimental procedures and characterizations**
5. **References**

1. Supplementary Figures and Figure Legends

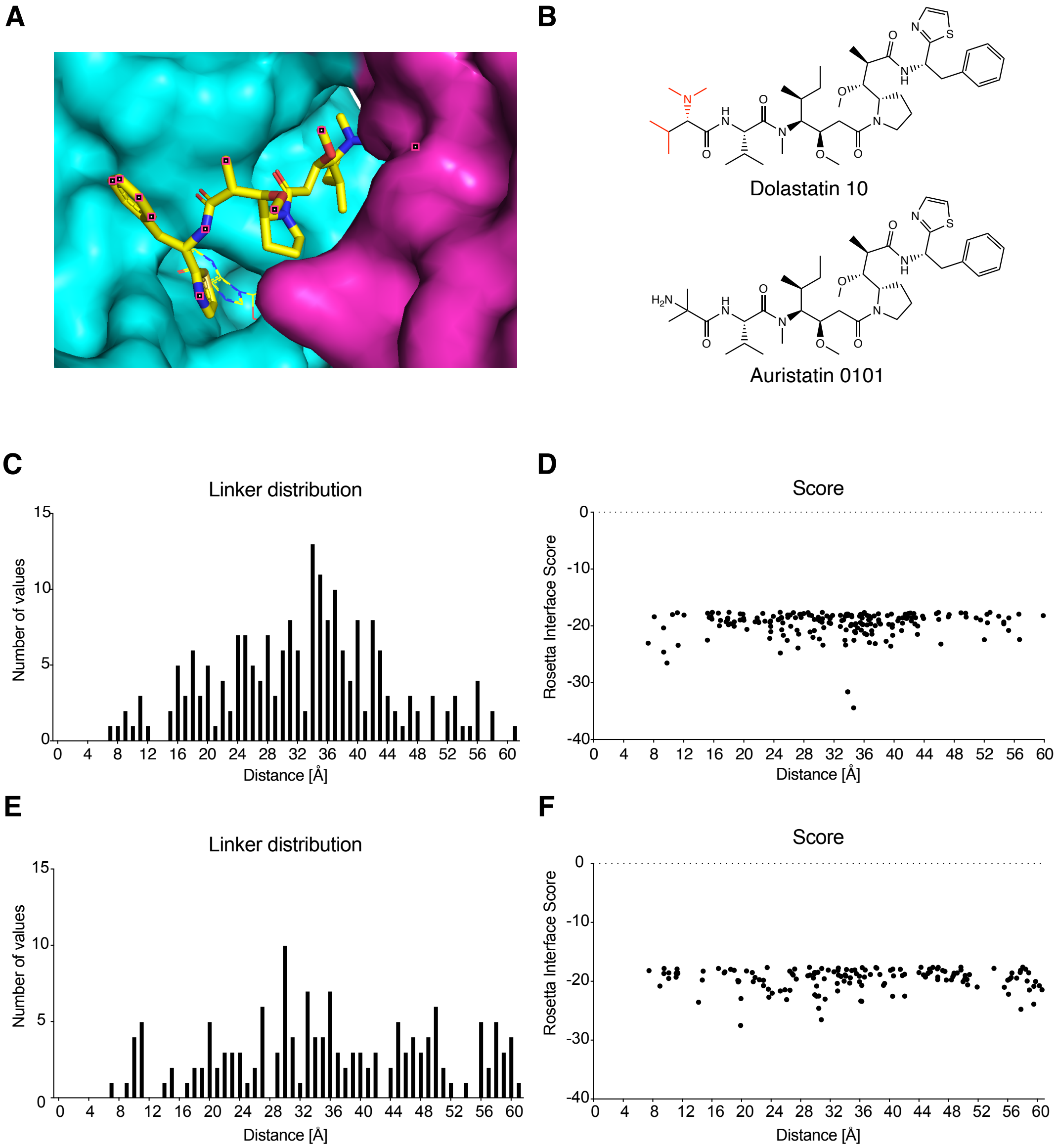

Supplementary Figure S1. Analysis of linker length and score of top 200 docked poses for CRBN and tubulin. (A) Atom selection on auristatin 0101 (PDB ID 4x1i). **(B)** Chemical structures of dolastatin 10 and auristatin 0101. Difference between the two compounds highlighted in red. **(C)** Distribution of linker distances for auristatin 0101 and lenalidomide in top 200 (by I_sc) docked poses calculated in Rosetta framework. Distances are calculated between lenalidomide and tubulin ligand. **(D)** Best 200 protein interface scores (I_sc) calculated in Rosetta framework for each of the poses. Data corresponds to linker distances calculated in (C). Each dot represents an indyvidual docked pose. **(E)** as in (C) for combretastatin A4 (PDB ID 5lyj). All atoms in combrestatin A4 were considered for linker calculation. **(F)** As in (D) corresponding to poses in (E).

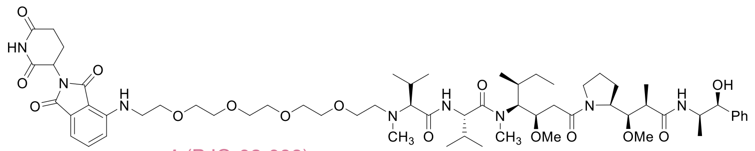

1 (BJG-02-098)

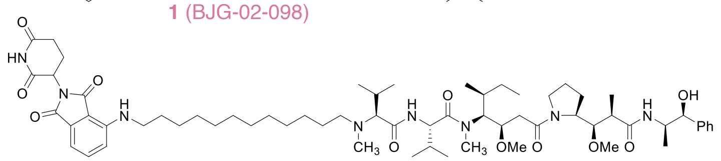

2 (BJG-02-108)

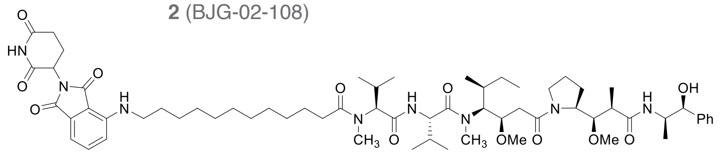

3 (BJG-02-095)

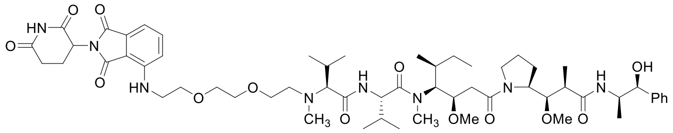

4 (BJG-02-088)

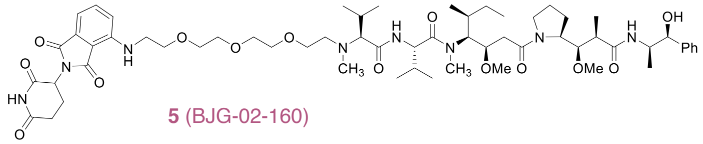

5 (BJG-02-160)

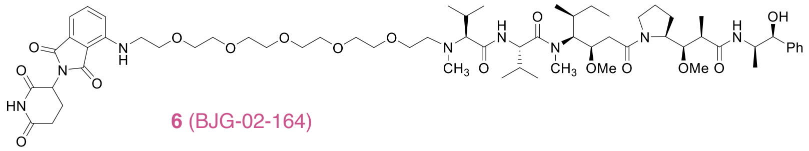

6 (BJG-02-164)

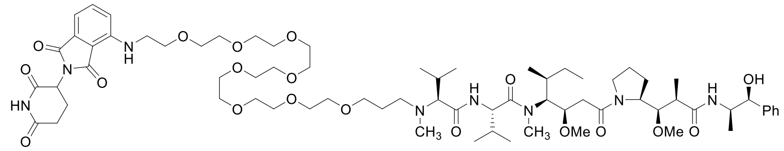

7 (BJG-03-089)

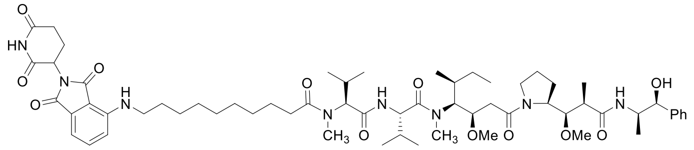

8 (BJG-02-097)

Supplementary Figure S2. MMAE-CRBN compounds. Structural formulae of the MMAE-CRBN compounds.

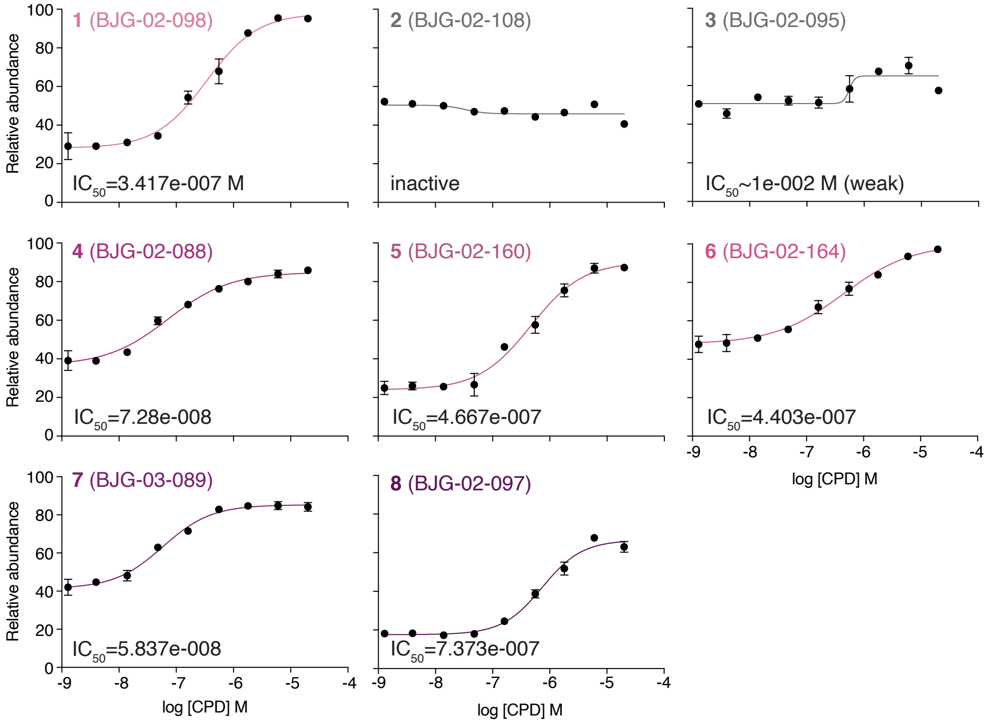

Supplementary Figure S3. MMAE-CRBN compounds engage CRBN in cells. Quantitative assessment of intracellular CRBN engagement using a $BRD4_{BRD2}$-eGFP-mCherry reporter assay. CRBN engagement was assessed by monitoring rescue of dBET6-mediated degradation of $BRD4_{BRD2}$-eGFP. Cells stably expressing $BRD4_{BRD2}$-eGFP and mCherry were treated with 100 nM of dBET6, a specific degrader of $BRD4_{BRD2}$, and increasing concentrations of the candidate tubulin degraders. The eGFP and mCherry signals were quantified by laser scanning cytometry and the concentration of compound that rescued 50% of $BRD4_{BRD2}$-eGFP fluorescence (EC_{50}) was determined by nonlinear regression. Data from n=2 biological replicates. Error bars represent standard deviation of the mean. CPD – compound with log base 10.

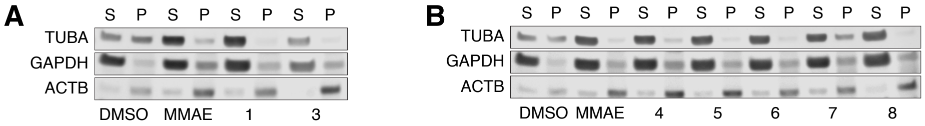

Supplementary Figure S4. MMAE-CRBN compounds destabilize microtubule cytoskeleton. (A and B) Biochemical partitioning of tubulin into soluble (S) and polymerized (P) in hTert-RPE1 cells across the indicated conditions. Note that treatment with 1 μM MMAE-CRBN derivatives for 5 h causes an increase in S and a decrease in P fraction of tubulin. Representative blots from three biological replicates.

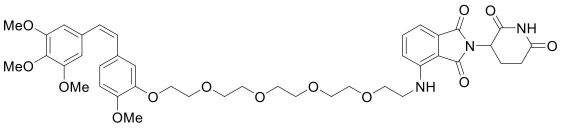

9 (BJG-03-081)

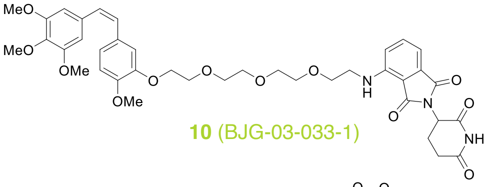

10 (BJG-03-033-1)

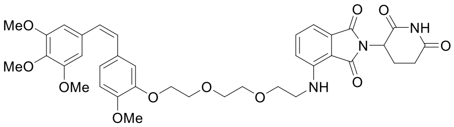

11 (BJG-02-158)

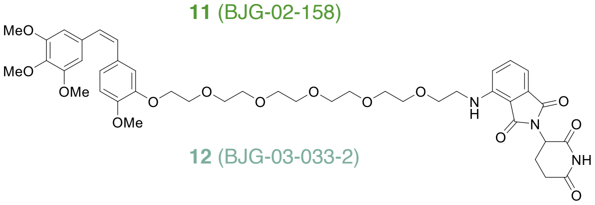

12 (BJG-03-033-2)

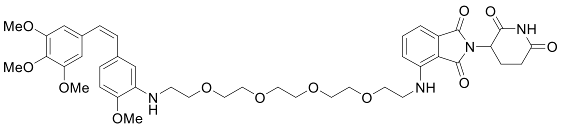

13 (BJG-03-144)

Supplementary Figure S5. CA4-CRBN compounds. Structural formulae of the CA4-CRBN compounds.

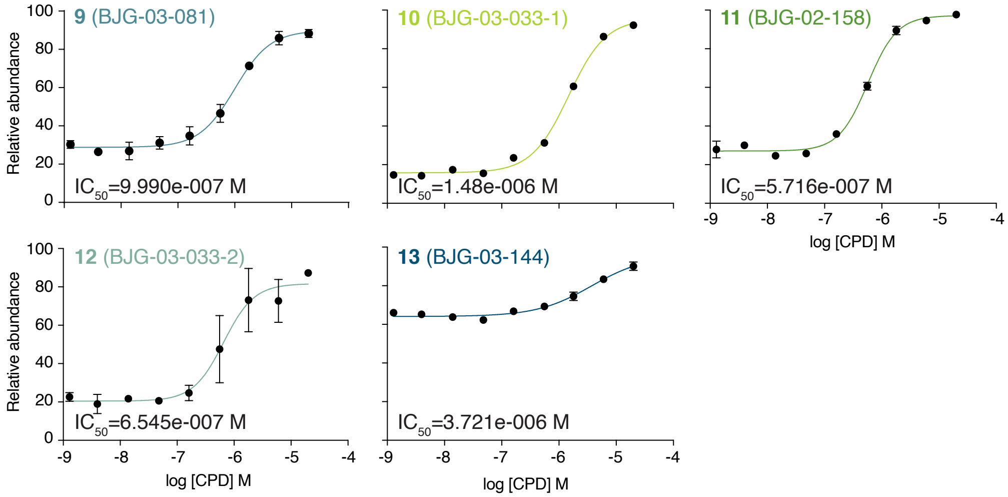

Supplementary Figure S6. CA4-CRBN compounds engage CRBN in cells. Quantitative assessment of intracellular CRBN engagement using a $BRD4_{BRD2}$-eGFP-mCherry reporter assay. CRBN engagement was assessed by monitoring rescue of dBET6-mediated degradation of $BRD4_{BRD2}$-eGFP. Cells stably expressing $BRD4_{BRD2}$-eGFP and mCherry were treated with 100 nM of dBET6, a specific degrader of $BRD4_{BRD2}$, and increasing concentrations of the candidate tubulin degraders. The eGFP and mCherry signals were quantified by laser scanning cytometry and the concentration of compound that rescued 50% of $BRD4_{BRD2}$-eGFP fluorescence (EC_{50}) was determined by nonlinear regression. Data from n=2 biological replicates. Error bars represent standard deviation of the mean. CPD – compound with log base 10.

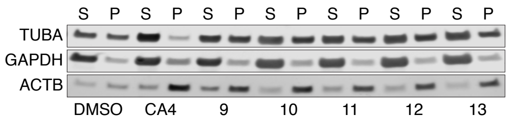

Supplementary Figure S7. CA4-CRBN compounds lack microtubule-destabilizing activity. Biochemical partitioning of tubulin into soluble (S) and polymerized (P) form in hTert-RPE1 cells across the indicated conditions. Note that the P fraction of tubulin remains present upon treatment with 1 μM CA4-CRBN derivatives for 5 h. Representative blots from three biological replicates.

2. General methods

Analytical grade solvents and commercially available reagents were purchased from commercial sources and used directly without further purification unless otherwise stated. Thin-layer chromatography (TLC) was carried out on Merck 60 F_{254} precoated, glass silica plates which were visualized with either ultraviolet light or stained with $KMnO_4$. Experiments were conducted under ambient conditions unless otherwise stated. ^{1}H-NMR, ^{13}C-NMR, and ^{19}F-NMR spectra were recorded at room temperature using a Bruker 500 (^{1}H-NMR at 500 MHz, ^{13}C-NMR at 125 MHz, and ^{19}F-NMR at 471 MHz). Chemical shifts are reported in ppm with reference to solvent signals [^{1}H-NMR: $CDCl_3$ (7.26 ppm), DMSO-d_6 (2.50 ppm); ^{13}C-NMR: $CDCl_3$ (77.16 ppm), DMSO-d_6 (39.52 ppm)]. Signal patterns are indicated as s, singlet; br s, broad singlet; d, doublet; t, triplet, q, quartet; p, pentet; and m, multiplet. Mass spectrometry (MS) analysis was obtained on a Waters Acquity UPLC-MS system using electrospray ionization (ESI) in positive ion mode, reporting the molecular ion $[M+H]^+$, $[M+Na]^+$, or a suitable fragment ion. Flash chromatography purification was conducted using an ISCO CombiFlash RF+ with RediSep Rf silica cartridges. Preparative reverse-phase HPLC purification was conducted using a Waters model 2545 pump and 2489 UV/Vis detector using SunFire Prep C18 5 μm columns (18x100 mm, 20 mL/min flow rate; 30x250 mm, 40 mL/min flow rate), and a gradient solvent system of water (0.035% TFA)/methanol (0.035% TFA) or water (0.035% TFA)/acetonitrile (0.035% TFA).

Abbreviations Used: CA4, combretastatin A-4; DCC, dicyclohexylcarbodiimide; DCM, dichloromethane; DIAD, diisopropyl azodicarboxylate; DIPEA, diisopropylethylamine; DMF, N,N-dimethylformamide; DMP, Dess-Martin periodinane; DMSO, dimethyl sulfoxide; EtOAc, ethyl acetate; HATU, O-(7-Azabenzotriazol-1-yl)-N,N,N',N'-tetramethyluronium hexafluorophosphate; HPLC, high-performance liquid chromatography; MeOH, methanol; MeCN, acetonitrile; MMAE, monomethyl auristatin E; TFA, trifluoroacetic acid; thal, thalidomide; UPLC-MS, ultra-performance liquid chromatography-tandem mass spectrometry.

3. Competitive displacement assay for cellular CRBN engagement

Cells stably expressing the $BRD4_{BD2}$-GFP with mCherry reporter [1] were seeded at 30-50% confluency in 384-well plates with 50 μL FluoroBrite DMEM media (Thermo Fisher Scientific A18967) containing 10% FBS per well a day before compound treatment. Compounds and 100 nM dBET6 were dispensed using a D300e Digital Dispenser (HP), normalized to 0.5% DMSO, and incubated with cells for 5 hours. The assay plate was imaged immediately using an Acumen High Content Imager (TTP Labtech) with 488 nm and 561 nm lasers in 2 μm x 1 μm grid per well format. The resulting images were analyzed using CellProfiler [2]. A series of image analysis steps ('image analysis pipeline') was constructed. First, the red and green channels were aligned and cropped to target the middle of each well (to avoid analysis of heavily clumped cells at the edges), and a background illumination function was calculated for both red and green channels of each well individually and subtracted to correct for illumination variations across the 384-well plate from various sources of error. An additional step was then applied to the green channel to suppress the analysis of large auto fluorescent artifacts and enhance the analysis of cell specific fluorescence by way of selecting for objects under a given size, 30 A.U., and with a given shape, speckles. mCherry-positive cells were then identified in the red channel filtering for objects between 8-60 pixels in diameter and using intensity to distinguish between clumped objects. The green channel was then segmented into GFP positive and negative areas and objects were labeled as GFP positive if at least 40% of it overlapped with a GFP positive area. The fraction of GFP-positive cells/mCherry-positive cells in each well was then calculated, and the green and red images were rescaled for visualization. The values for the concentrations that lead to a 50% increase in $BRD4_{BD2}$-eGFP accumulation (EC_{50}) were calculated using the nonlinear fit variable slope model (GraphPad Software).

4. Experimental procedures and characterizations

General procedure A. S_NAr reaction of primary amines with **S1.**
A solution of aryl fluoride **S1** (1.0 equiv), primary amine (1.0-1.3 equiv), and DIPEA (2.0-4.0 equiv) in DMSO (0.1-0.3 M) was heated to 130 °C overnight. The reaction was then cooled to room temperature, diluted with water, and extracted with ethyl acetate. The combined organic layers were washed three times with water and then with brine, dried over magnesium sulfate, filtered, and concentrated.

General procedure B. Amide coupling with MMAE.
To a solution of thal-linker-CO_2H (1.5 equiv) and monomethyl auristatin E (1.0 equiv) in DMSO (0.05 M) were added HATU (1.2 equiv) and DIPEA (2.5-3.5 equiv). The reaction was stirred at room temperature for 4 hours, and then diluted with DMSO and purified directly by reverse-phase preparative HPLC.

General procedure C. Reductive amination with MMAE.
To solution of thal-linker-OH (2.0 equiv relative to MMAE) in DCM (0.05 M) was added Dess-Martin periodinane (3.0-4.0 equiv relative to MMAE). The mixture was stirred at room temperature overnight, and then filtered and concentrated with a stream of nitrogen. The crude aldehyde intermediate was dissolved in methanol (0.05 M). Monomethyl auristatin E (1.0 equiv) was added, followed by sodium cyanoborohydride ($NaBH_3CN$; 3.3 equiv) and 2 drops of glacial acetic acid. The reaction was stirred at room temperature overnight. Methanol was removed with a stream of N_2, and then the crude reaction was diluted with DMSO and purified by reverse-phase preparative HPLC.

General procedure D. Mitsunobu reaction with CA4.
To a solution of CA4 (1.0 equiv), triphenylphosphine (1.0 equiv), and N-Boc-linker-alcohol (1.0 equiv) in DMF (0.4 M), DIAD (1.0 equiv) was added at room temperature. The reaction was stirred overnight, then

diluted with water and extracted three times with DCM. The combined organic layers were washed with 1 M hydrochloric acid, saturated aqueous sodium bicarbonate, and brine, then dried over magnesium sulfate, filtered, and concentrated.

2-(2,6-dioxopiperidin-3-yl)-4-fluoroisoindoline-1,3-dione (**S1**)

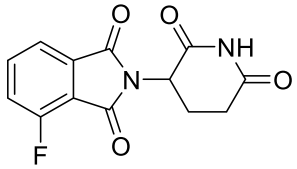

A suspension of 3-fluorophthalic anhydride (338 mg, 2.0 mmol, 1.0 equiv), potassium acetate (616 mg, 6.2 mmol, 3.1 equiv), and 3-aminopiperidine-2,6-dione hydrochloride (363 mg, 2.2 mmol, 1.1 equiv) in glacial acetic acid (6 mL) was heated to 120 °C overnight. The reaction was cooled to room temperature and concentrated by rotary evaporation. The residue was taken up in 100 mL water and extracted with DCM (4x50 mL). The combined organic layers were washed with brine, dried over $MgSO_4$, filtered and concentrated to provide **S1** (505 mg, 91% yield) as a beige powder which was used directly without purification.

^{1}H-NMR (500 MHz, DMSO-d_6) δ 11.14 (s, 1H), 7.95 (ddd, *J* = 8.5, 7.4, 4.4 Hz, 1H), 7.79 (d, *J* = 7.3 Hz, 1H), 7.73 (t, *J* = 8.9 Hz, 1H), 5.16 (dd, *J* = 12.9, 5.4 Hz, 1H), 2.89 (ddd, *J* = 17.1, 13.9, 5.4 Hz, 1H), 2.66 – 2.57 (m, 1H), 2.57 – 2.52 (m, 1H), 2.12 – 2.01 (m, 1H).

^{19}F NMR (471 MHz, DMSO-d_6) δ -114.67 (dd, *J* = 9.8, 4.7 Hz).

2-(2,6-dioxopiperidin-3-yl)-4-((14-hydroxy-3,6,9,12-tetraoxatetradecyl)amino)isoindoline-1,3-dione (**S2**)

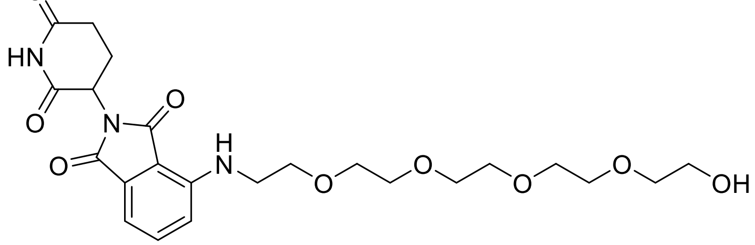

The title compound was prepared according to General Procedure A using **S1** (55.1 mg, 0.20 mmol, 1.0 equiv), amino-PEG5-alcohol (56.0 mg, 0.24 mmol, 1.2 equiv), and DIPEA (70.0 μL, 0.40 mmol, 2.0 equiv) in DMSO (0.60 mL) at heated to 100 °C overnight. ISCO flash chromatography (12 g silica, 5 – 20% MeOH/DCM, 5 minute gradient then 3 min isocratic hold) provided **S2** (19.5 mg, 20% yield) as a sticky green solid.

^{1}H NMR (500 MHz, $CDCl_3$) δ 8.52 (s, 1H), 7.49 (dd, *J* = 8.5, 7.1 Hz, 1H), 7.10 (d, *J* = 7.1 Hz, 1H), 6.92 (d, *J* = 8.5 Hz, 1H), 4.91 (dd, *J* = 12.2, 5.4 Hz, 1H), 3.75 – 3.70 (m, 4H), 3.70 – 3.63 (br s, 13H), 3.63 – 3.58 (m, 3H), 3.47 (t, *J* = 5.4 Hz, 2H), 2.91 – 2.84 (m, 1H), 2.81 – 2.67 (m, 2H), 2.18 – 2.07 (m, 1H).

MS (ESI) *m/z*: 494.26 $(M+H)^+$.

(2S)-17-((2-(2,6-dioxopiperidin-3-yl)-1,3-dioxoisoindolin-4-yl)amino)-N-((S)-1-(((3R,4S,5S)-1-((S)-2-((1R,2R)-3-(((1S,2R)-1-hydroxy-1-phenylpropan-2-yl)amino)-1-methoxy-2-methyl-3-oxopropyl)pyrrolidin-1-yl)-3-methoxy-5-methyl-1-oxoheptan-4-yl)(methyl)amino)-3-methyl-1-oxobutan-2-yl)-2-isopropyl-3-methyl-6,9,12,15-tetraoxa-3-azaheptadecanamide (**1, BJG-02-098**)

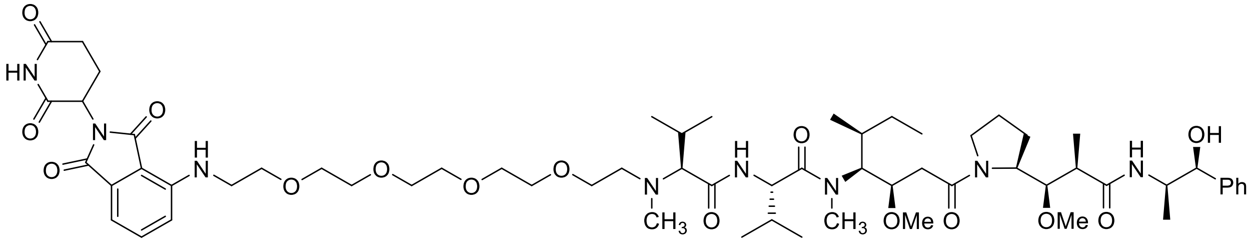

The title compound was prepared according to General Procedure C using **S2** (9.7 mg, 0.020 mmol) and DMP (16.5 mg, 0.039 mmol) in 0.40 mL DCM for the oxidation step; monomethyl auristatin E (7.7 mg, 0.011 mmol, 1.0 equiv) and $NaBH_3CN$ (2.3 mg, 0.036 mmol, 3.3 equiv) in MeOH (0.22 mL) were used for the reductive amination step. Reverse-phase prep HPLC (100 to 40% H_2O/MeCN, 20 mL/min, 45 min) followed by lyophilization from H_2O/MeCN provided **1** (4.7 mg TFA salt, 33% yield) as a yellow powder.

^1H NMR (500 MHz, DMSO-d_6) δ 11.09 (s, 1H), 9.64 – 9.22 (m, 1H), 8.91 (s, 1H), 7.67 – 7.53 (m, 1H), 7.31 (d, *J* = 7.6 Hz, 2H), 7.26 (t, *J* = 7.5 Hz, 2H), 7.20 – 7.11 (m, 2H), 7.05 (d, *J* = 7.1 Hz, 1H), 6.60 (t, *J* = 5.8 Hz, 1H), 5.55 – 5.23 (m, 1H), 5.05 (dd, *J* = 12.8, 5.5 Hz, 1H), 4.74 (d, *J* = 11.1 Hz, 1H), 4.62 (t, *J* = 8.4 Hz, 1H), 4.46 (dd, *J* = 22.7, 6.3 Hz, 1H), 4.05 – 3.96 (m, 2H), 3.62 (t, *J* = 5.4 Hz, 3H), 3.60 – 3.50 (m, 17H), 3.25 (s, 3H), 3.23 (s, 1H), 3.20 (s, 2H), 3.17 (s, 1H), 3.13 (s, 2H), 3.05 (dt, *J* = 11.3, 7.0 Hz, 1H), 2.99 (s, 1H), 2.93 – 2.73 (m, 4H), 2.63 – 2.55 (m, 1H), 2.42 (dd, *J* = 15.7, 8.4 Hz, 1H), 2.27 (td, *J* = 14.6, 12.0, 6.9 Hz, 2H), 2.17 – 2.08 (m, 1H), 2.08 – 1.95 (m, 2H), 1.89 – 1.66 (m, 3H), 1.60 – 1.38 (m, 2H), 1.36 – 1.25 (m, 2H), 1.04 (d, *J* = 6.7 Hz, 2H), 1.02 – 0.84 (m, 21H), 0.77 (dt, *J* = 9.7, 7.4 Hz, 3H).

MS (ESI) *m/z*: 1193.78 $(M+H)^+$.

2-(2,6-dioxopiperidin-3-yl)-4-((12-hydroxydodecyl)amino)isoindoline-1,3-dione (**S3**)

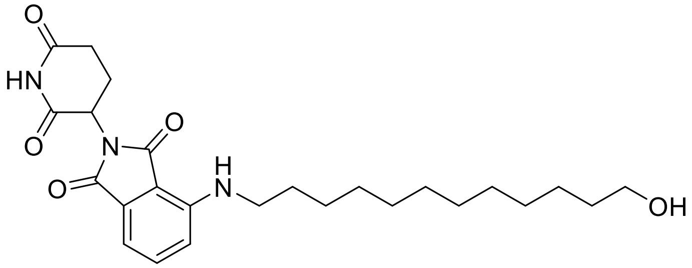

The title compound was prepared according to General Procedure A using **S1** (58.4 mg, 0.20 mmol, 1.0 equiv), 12-aminododecanol (51.4 mg, 0.24 mmol, 1.2 equiv), and DIPEA (70.0 μL, 0.40 mmol, 2.0 equiv) in DMSO (0.60 mL) at 130 °C for 3 hours, providing **S3** (111 mg, quantitative yield, contains residual solvent) as a green solid which was used directly without purification.

^1H NMR (500 MHz, $CDCl_3$) δ 8.03 (s, 1H), 7.49 (dd, *J* = 8.3, 7.0 Hz, 1H), 7.08 (d, *J* = 7.1 Hz, 1H), 6.88 (d, *J* = 8.5 Hz, 1H), 6.23 (s, 1H), 4.91 (dd, *J* = 12.2, 5.3 Hz, 1H), 3.67 – 3.61 (m, 4H), 3.25 (t, *J* = 6.5 Hz, 2H), 2.94 – 2.67 (m, 3H), 2.16 – 2.09 (m, 1H), 1.71 – 1.60 (m, 4H), 1.61 – 1.48 (m, 4H), 1.46 – 1.37 (m, 4H), 1.37 – 1.27 (m, 6H).

MS (ESI) *m/z*: 458.38 $(M+H)^+$.

(2S)-2-((2S)-2-((12-((2-(2,6-dioxopiperidin-3-yl)-1,3-dioxoisoindolin-4-yl)amino)dodecyl)(methyl)amino)-3-methylbutanamido)-N-((3R,4S,5S)-1-((S)-2-((1R,2R)-3-(((1S,2R)-1-hydroxy-1-phenylpropan-2-yl)amino)-1-methoxy-2-methyl-3-oxopropyl)pyrrolidin-1-yl)-3-methoxy-5-methyl-1-oxoheptan-4-yl)-N,3-dimethylbutanamide (**2, BJG-02-108**)

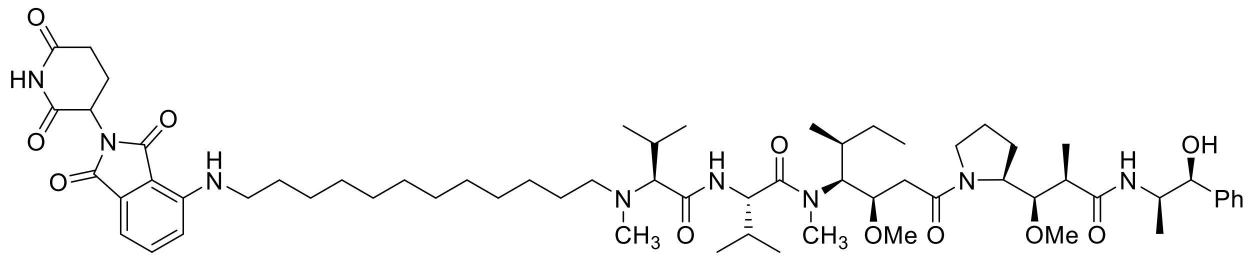

The title compound was prepared according to General Procedure C using **S3** (10.8 mg, 0.020 mmol) and DMP (13.5 mg, 0.030 mmol) in 0.30 mL DCM for the oxidation step; monomethyl auristatin E (7.5 mg, 0.010 mmol, 1.0 equiv) and $NaBH_3CN$ (2.9 mg, 0.046 mmol, 4.6 equiv) in MeOH (0.30 mL) were used for the reductive amination step. Reverse-phase prep HPLC (80 to 0% H_2O/MeCN, 20 mL/min, 45 min) followed by lyophilization from H_2O/MeCN provided **2** (3.9 mg TFA salt, 30% yield) as a yellow powder.

^{1}H NMR (500 MHz, DMSO-d_6) δ 11.09 (s, 1H), 9.65 – 9.29 (rotamers, 2 singlets, 1H), 8.90 (rotamers, 2 doublets, *J* = 8.5 Hz, 1H), 7.92 (t, *J* = 7.8 Hz, 1H), 7.57 (t, *J* = 8.3 Hz, 1H), 7.31 (dd, *J* = 7.7, 4.2 Hz, 2H), 7.26 (t, *J* = 7.5 Hz, 2H), 7.16 (q, *J* = 7.1 Hz, 1H), 7.08 (dd, *J* = 8.7, 3.8 Hz, 1H), 7.02 (d, *J* = 7.0 Hz, 1H), 6.51 (t, *J* = 6.3 Hz, 1H), 5.44 (br s, 1H), 5.04 (dd, *J* = 12.8, 5.4 Hz, 1H), 4.75 (d, *J* = 11.6 Hz, 1H), 4.69 – 4.53 (m, 1H), 4.52 – 4.39 (m, 1H), 4.07 – 3.90 (m, 2H), 3.90 – 3.81 (m, 1H), 3.63 – 3.53 (m, 2H), 3.35 – 3.24 (m, 7H), 3.23 (s, 2H), 3.20 (s, 2H), 3.17 (s, 1H), 3.16 – 3.11 (m, 2H), 3.10 – 2.93 (m, 4H), 2.92 – 2.72 (m, 4H), 2.59 (dt, *J* = 19.4, 3.7 Hz, 1H), 2.43 (dd, *J* = 15.7, 8.0 Hz, 1H), 2.34 – 2.20 (m, 2H), 2.13 (tt, *J* = 8.9, 7.7 Hz, 1H), 2.09 – 1.94 (m, 2H), 1.91 – 1.66 (m, 4H), 1.62 – 1.51 (m, 4H), 1.38 – 1.18 (m, 20H), 1.05 (d, *J* = 6.6 Hz, 2H), 1.03 – 0.83 (m, 23H), 0.82 – 0.71 (m, 4H).

MS (ESI) *m/z*: 1157.87 (M+H)$^+$.

12-((2-(2,6-dioxopiperidin-3-yl)-1,3-dioxoisoindolin-4-yl)amino)dodecanoic acid (**S4**)

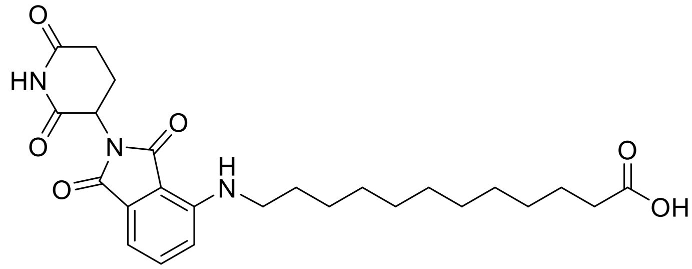

The title compound was prepared according to General Procedure A using **S1** (56.1 mg, 0.20 mmol, 1.0 equiv), 12-aminododecanoic acid (52.8 mg, 0.24 mmol, 1.2 equiv), and DIPEA (70.0 µL, 0.40 mmol, 2.0 equiv) in DMSO (0.60 mL) at 130 °C for 3 hours. ISCO flash chromatography (4 g silica, 0 – 10% MeOH/DCM, 4 minute gradient then 2 minute isocratic hold) provided **S4** (44.6 mg, 47% yield) as a green film.

^{1}H NMR (500 MHz, DMSO-d_6) δ 7.55 (t, *J* = 8.1, 7.6 Hz, 1H), 7.04 (d, *J* = 8.6 Hz, 1H), 6.99 (d, *J* = 7.0 Hz, 1H), 6.46 (t, *J* = 5.9 Hz, 1H), 5.03 (dd, *J* = 12.9, 5.4 Hz, 1H), 2.94 – 2.81 (m, 1H), 2.64 – 2.52 (m, 2H), 2.16 (t, *J* = 7.4 Hz, 2H), 2.07 – 1.95 (m, 1H), 1.58 (p, *J* = 7.2 Hz, 2H), 1.48 (p, *J* = 7.3 Hz, 2H), 1.40 – 1.16 (m, 16H).

MS (ESI) *m/z*: 472.31 (M+H)$^+$.

12-((2-(2,6-dioxopiperidin-3-yl)-1,3-dioxoisoindolin-4-yl)amino)-N-((S)-1-(((S)-1-(((3R,4S,5S)-1-((S)-2-((1R,2R)-3-(((1S,2R)-1-hydroxy-1-phenylpropan-2-yl)amino)-1-methoxy-2-methyl-3-oxopropyl)pyrrolidin-1-yl)-3-methoxy-5-methyl-1-oxoheptan-4-yl)(methyl)amino)-3-methyl-1-oxobutan-2-yl)amino)-3-methyl-1-oxobutan-2-yl)-N-methyldodecanamide (**3, BJG-02-095**)

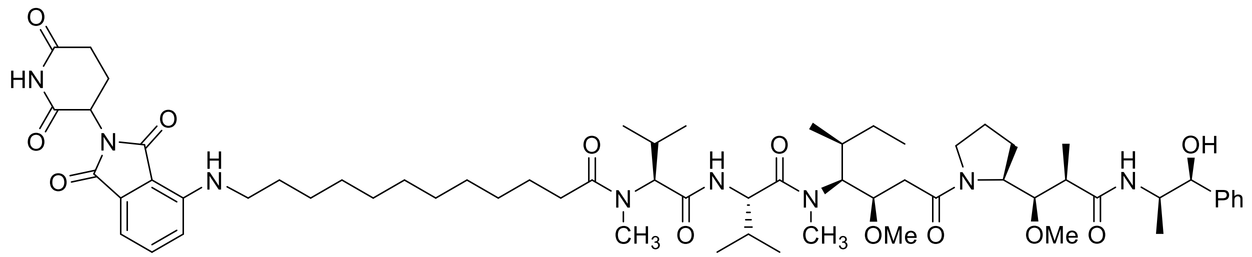

The title compound was prepared according to General Procedure B using **S4** (8.1 mg, 0.0172 mmol, 1.55 equiv), monomethyl auristatin E (7.7 mg, 0.0110 mmol, 1.0 equiv), HATU (5.3 mg, 0.0132 mmol, 1.2 equiv) and DIPEA (5.00 μL, 0.0275 mmol, 2.5 equiv) in 0.22 mL DMSO. The reaction was stirred at room temperature for 4 hours. Reverse-phase prep HPLC (80 to 0% H_2O/MeCN, 20 mL/min, 45 min) followed by lyophilization from H_2O/MeCN provided **3** (8.2 mg, TFA salt, 58% yield) as a fluffy yellow powder.
^{1}H NMR (500 MHz, DMSO-*d*$_6$) (Methoxy groups hidden under water peak) δ 11.08 (s, 1H), 8.59 – 8.49 (rotamer, m, 0.5H), 7.91 – 7.84 (rotamer, m, 0.5H), 7.72 – 7.60 (m, 1H), 7.57 (t, *J* = 7.8 Hz, 1H), 7.30 (d, *J* = 7.0 Hz, 2H), 7.28 – 7.22 (m, 2H), 7.21 – 7.12 (m, 1H), 7.08 (d, *J* = 8.6 Hz, 1H), 7.01 (d, *J* = 7.0 Hz, 1H), 6.51 (t, *J* = 6.3 Hz, 1H), 5.05 (dd, *J* = 12.7, 5.4 Hz, 1H), 4.78 – 4.57 (m, 1H), 4.56 – 4.46 (m, 1H), 4.46 – 4.37 (m, 1H), 4.08 – 3.91 (m, 3H), 3.63 – 3.53 (m, 2H), 3.34 – 3.21 (m, 8H), 3.18 (dd, *J* = 11.4, 4.4 Hz, 3H), 3.11 (d, *J* = 9.8 Hz, 2H), 2.97 (d, *J* = 11.4 Hz, 1H), 2.94 – 2.81 (m, 4H), 2.63 – 2.54 (m, 1H), 2.45 – 2.22 (m, 4H), 2.19 – 2.07 (m, 2H), 2.05 – 1.98 (m, 2H), 1.86 – 1.66 (m, 3H), 1.61 – 1.40 (m, 7H), 1.37 – 1.29 (m, 5H), 1.07 – 0.95 (m, 7H), 0.91 – 0.68 (m, 20H).
MS (ESI) *m/z*: 1171.87 $(M+H)^+$.

2-(2,6-dioxopiperidin-3-yl)-4-((2-(2-(2-hydroxyethoxy)ethoxy)ethyl)amino)isoindoline-1,3-dione (**S5**)

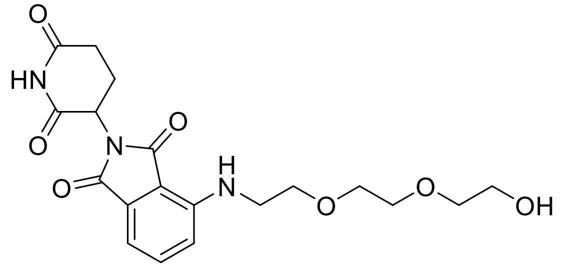

The title compound was prepared according to General Procedure A using **S1** (55.7 mg, 0.20 mmol, 1.0 equiv), amino-PEG3-alcohol (51.3 mg, 0.34 mmol, 1.7 equiv), and DIPEA (0.10 mL, 0.60 mmol, 3.0 equiv) in DMSO (1.0 mL) at 120 °C for 4 hours. ISCO flash chromatography (4 g silica, 0 – 10% MeOH/DCM, 4 minute gradient then 5 minute isocratic hold) provided **S5** (43.0 mg, 53% yield) as a green oil.
^{1}H NMR (500 MHz, DMSO-*d*$_6$) δ 11.08 (s, 1H), 7.58 (dd, *J* = 8.6, 7.0 Hz, 1H), 7.15 (d, *J* = 8.6 Hz, 1H), 7.04 (d, *J* = 7.0 Hz, 1H), 6.60 (t, *J* = 5.8 Hz, 1H), 5.05 (dd, *J* = 12.7, 5.4 Hz, 1H), 4.54 (t, *J* = 5.7 Hz, 1H), 3.62 (t, *J* = 5.6 Hz, 2H), 3.58 – 3.52 (m, 4H), 3.49 – 3.44 (m, 4H), 3.44 – 3.39 (m, 2H), 2.88 (ddd, *J* = 16.8, 13.7, 5.4 Hz, 1H), 2.63 – 2.52 (m, 2H), 2.03 (dtd, *J* = 12.9, 5.1, 2.0 Hz, 1H). MS (ESI) *m/z*: 406.27 $(M+H)^+$.

(2S)-2-((2S)-2-((2-(2-(2-((2-(2,6-dioxopiperidin-3-yl)-1,3-dioxoisoindolin-4-yl)amino)ethoxy)ethoxy)ethyl)(methyl)amino)-3-methylbutanamido)-N-((3R,4S,5S)-1-((S)-2-((1R,2R)-3-(((1S,2R)-1-hydroxy-1-phenylpropan-2-yl)amino)-1-methoxy-2-methyl-3-oxopropyl)pyrrolidin-1-yl)-3-methoxy-5-methyl-1-oxoheptan-4-yl)-N,3-dimethylbutanamide (**4, BJG-03-088**)

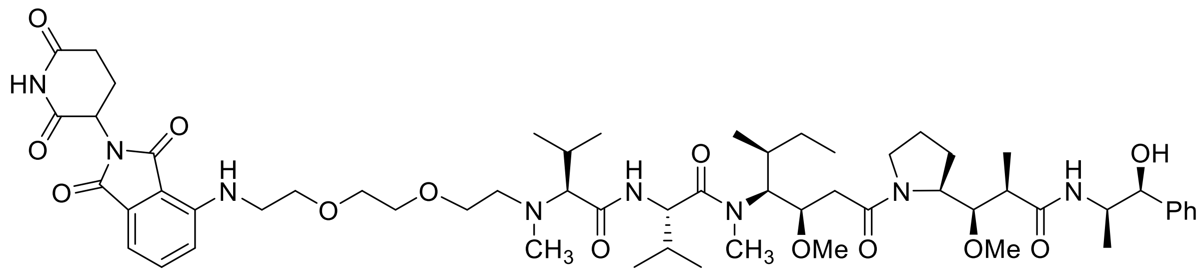

The title compound was prepared according to General Procedure C using **S5** (9.8 mg, 0.025 mmol) and DMP (19.7 mg, 0.046 mmol) in 0.50 mL DCM for the oxidation step; monomethyl auristatin E (7.1 mg, 0.010 mmol, 1.0 equiv) and $NaBH_3CN$ (3.0 mg, 0.047 mmol, 4.7 equiv) in MeOH (0.30 mL) were used for the reductive amination step. Reverse-phase prep HPLC (100 to 40% H_2O/MeCN, 20 mL/min, 45 min) followed by lyophilization from H_2O/MeCN provided **4** (4.2 mg TFA salt, 34% yield) as a yellow powder.
^{1}H NMR (500 MHz, DMSO-d_6) δ 11.09 (s, 1H), 9.65 – 9.27 (m, 1H), 8.90 (s, 1H), 7.92 (d, *J* = 8.6 Hz, 1H), 7.59 (dd, *J* = 8.6, 7.1 Hz, 1H), 7.30 (d, *J* = 7.0 Hz, 2H), 7.29 – 7.22 (m, 3H), 7.18 (d, *J* = 7.1 Hz, 1H), 7.17 – 7.12 (m, 2H), 7.05 (d, *J* = 7.3 Hz, 1H), 6.60 (br s, 1H), 5.05 (dd, *J* = 12.8, 5.4 Hz, 1H), 4.74 (br s, 1H), 4.63 (t, *J* = 8.4 Hz, 1H), 4.46 (rotamers, 2 doublets, *J* = 6.3 Hz, 1H), 4.04 – 3.96 (m, 3H), 3.68 – 3.52 (m, 12H), 3.52 – 3.43 (m, 3H), 3.25 (s, 2H), 3.23 (s, 1H), 3.20 (s, 3H), 3.17 (s, 1H), 3.13 (s, 2H), 2.99 (s, 1H), 2.93 – 2.73 (m, 4H), 2.63 – 2.55 (m, 1H), 2.44 – 2.37 (m, 1H), 2.33 – 2.22 (m, 2H), 2.16 – 2.07 (m, 1H), 2.07 – 1.94 (m, 2H), 1.89 – 1.67 (m, 3H), 1.60 – 1.36 (m, 2H), 1.34 – 1.21 (m, 2H), 1.09 – 0.70 (m, 28H).
MS (ESI) *m/z*: 1105.65 (M+H)$^+$.

2-(2,6-dioxopiperidin-3-yl)-4-((2-(2-(2-(2-hydroxyethoxy)ethoxy)ethoxy)ethyl)amino)isoindoline-1,3-dione (**S6**)

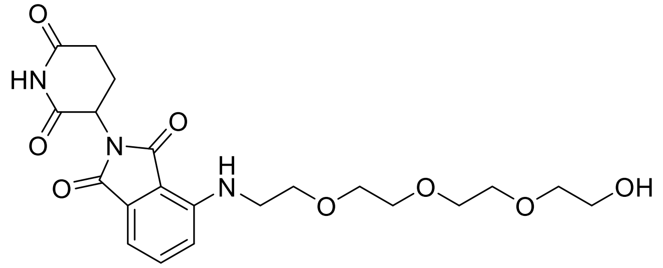

The title compound was prepared according to General Procedure A using **S1** (70.0 mg, 0.25 mmol, 1.0 equiv), amino-PEG4-alcohol (63.0 mg, 0.33 mmol, 1.3 equiv), and DIPEA (0.09 mL, 0.50 mmol, 2.0 equiv) in DMF (0.8 mL) at 120 °C for 2 hours. ISCO flash chromatography (12 g silica, 0 – 10% MeOH/DCM, 10 minute gradient then 3 minute isocratic hold) provided **S6** (25.4 mg, 22% yield) as a green oil.
^{1}H NMR (500 MHz, $CDCl_3$) δ 8.39 (s, 1H), 7.48 (dd, *J* = 8.5, 7.1 Hz, 1H), 7.10 (d, *J* = 7.1 Hz, 1H), 6.92 (d, *J* = 8.5 Hz, 1H), 6.51 (s, 1H), 4.99 – 4.83 (m, 1H), 3.76 – 3.70 (m, 4H), 3.70 – 3.64 (m, 8H), 3.63 – 3.58 (m, 2H), 3.47 (t, *J* = 5.4 Hz, 2H), 2.91 – 2.67 (m, 3H), 2.19 – 2.01 (m, 2H).
MS (ESI) *m/z*: 449.88 (M+H)$^+$.

(2S)-14-((2-(2,6-dioxopiperidin-3-yl)-1,3-dioxoisoindolin-4-yl)amino)-N-((S)-1-(((3R,4S,5S)-1-((S)-2-((1R,2R)-3-(((1S,2R)-1-hydroxy-1-phenylpropan-2-yl)amino)-1-methoxy-2-methyl-3-oxopropyl)pyrrolidin-1-yl)-3-methoxy-5-methyl-1-oxoheptan-4-yl)(methyl)amino)-3-methyl-1-oxobutan-2-yl)-2-isopropyl-3-methyl-6,9,12-trioxa-3-azatetradecanamide (**5, BJG-02-160**)

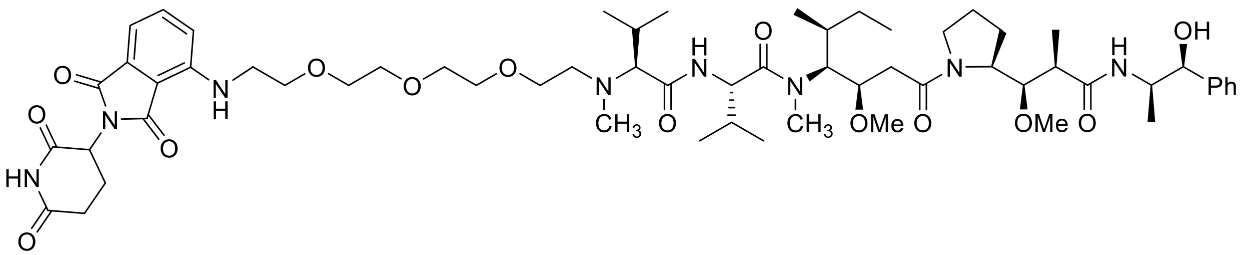

The title compound was prepared according to General Procedure C using **S6** (9.0 mg, 0.020 mmol) and DMP (12.7 mg, 0.030 mmol) in 0.40 mL DCM for the oxidation step; monomethyl auristatin E (7.3 mg, 0.010 mmol, 1.0 equiv) and $NaBH_3CN$ (2.4 mg, 0.038 mmol, 3.8 equiv) in MeOH (0.25 mL) were used for the reductive amination step. Reverse-phase prep HPLC (100 to 40% H_2O/MeCN, 20 mL/min, 45 min) followed by lyophilization from H_2O/MeCN provided **5** (4.7 mg TFA salt, 37% yield) as a yellow powder.

^{1}H NMR (500 MHz, DMSO-*d*$_6$) δ 11.08 (s, 1H), 8.04 (t, *J* = 10.0 Hz, 1H), 7.95 (s, 1H), 7.89 (rotamer, d, *J* = 8.7 Hz, 0.5H), 7.62 (rotamer, d, *J* = 8.5 Hz, 0.5H), 7.58 (dd, *J* = 8.5, 7.1 Hz, 1H), 7.36 – 7.22 (m, 4H), 7.20 – 7.10 (m, 2H), 7.03 (d, *J* = 7.0 Hz, 1H), 6.60 (t, *J* = 5.8 Hz, 1H), 5.38 (rotamers, 2 doublets, *J* = 4.4 Hz, 1H), 5.05 (dd, *J* = 12.7, 5.4 Hz, 1H), 4.79 – 4.39 (m, 3H), 4.07 – 3.90 (m, 2H), 3.65 – 3.38 (m, 17H), 3.25 (s, 2H), 3.23 (s, 1H), 3.19 (s, 2H), 3.17 (s, 1H), 3.14 (s, 2H), 3.08 – 3.01 (m, 1H), 2.98 (s, 1H), 2.89 (s, 3H), 2.74 – 2.64 (m, 4H), 2.62 – 2.55 (m, 1H), 2.54 (s, 3H), 2.41 (d, *J* = 16.1 Hz, 1H), 2.32 – 2.17 (m, 4H), 2.13 (ddd, *J* = 9.8, 6.8, 3.9 Hz, 1H), 2.07 – 1.65 (m, 7H), 1.60 – 1.41 (m, 2H), 1.36 – 1.11 (m, 3H), 1.05 (d, *J* = 6.6 Hz, 2H), 1.03 – 0.95 (m, 4H), 0.93 – 0.81 (m, 14H), 0.78 – 0.64 (m, 6H).

MS (ESI) *m/z*: 1148.76 (M+H)$^+$.

2-(2,6-dioxopiperidin-3-yl)-4-((17-hydroxy-3,6,9,12,15-pentaoxaheptadecyl)amino)isoindoline-1,3-dione (**S7**)

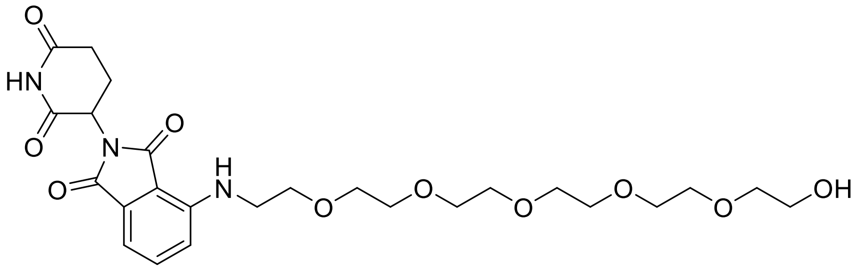

The title compound was prepared according to General Procedure A using **S1** (70.4 mg, 0.25 mmol, 1.0 equiv), amino-PEG6-alcohol (85.9 mg, 0.30 mmol, 1.2 equiv), and DIPEA (0.09 mL, 0.50 mmol, 2.0 equiv) in DMF (0.8 mL) at 120 °C for 2 hours. ISCO flash chromatography (12 g silica, 0 – 10% MeOH/DCM, 10 minute gradient then 3 minute isocratic hold) provided **S7** (27.7 mg, 21% yield) as a green oil.

^{1}H NMR (500 MHz, $CDCl_3$) δ 8.56 (s, 1H), 7.48 (dd, *J* = 8.5, 7.1 Hz, 1H), 7.09 (d, *J* = 7.1 Hz, 1H), 6.92 (d, *J* = 8.5 Hz, 1H), 6.50 (s, 1H), 5.58 – 5.18 (m, 1H), 4.98 – 4.82 (m, 1H), 3.75 – 3.69 (m, 6H), 3.69 – 3.58 (m, 12H), 3.53 (t, *J* = 5.1 Hz, 2H), 3.46 (t, *J* = 5.4 Hz, 2H), 3.31 (br s, 2H), 2.93 – 2.65 (m, 3H), 2.15 – 2.07 (m, 1H).

MS (ESI) *m/z*: 537.89 (M+H)$^+$.

(2S)-20-((2-(2,6-dioxopiperidin-3-yl)-1,3-dioxoisoindolin-4-yl)amino)-N-((S)-1-(((3R,4S,5S)-1-((S)-2-((1R,2R)-3-(((1S,2R)-1-hydroxy-1-phenylpropan-2-yl)amino)-1-methoxy-2-methyl-3-oxopropyl)pyrrolidin-1-yl)-3-methoxy-5-methyl-1-oxoheptan-4-yl)(methyl)amino)-3-methyl-1-oxobutan-2-yl)-2-isopropyl-3-methyl-6,9,12,15,18-pentaoxa-3-azaicosanamide (**6, BJG-02-164**)

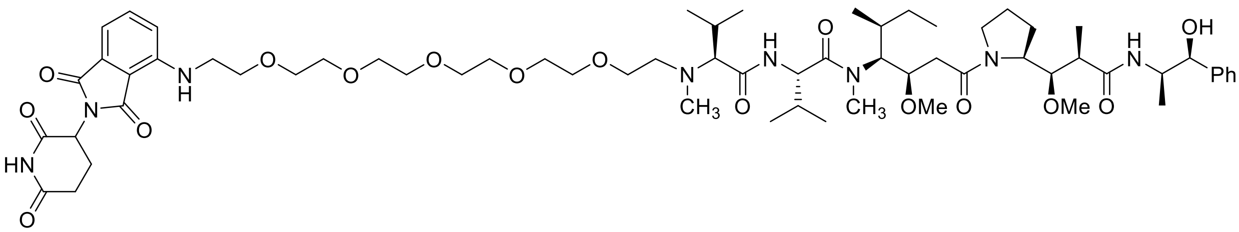

The title compound was prepared according to General Procedure C using **S7** (13.8 mg, 0.025 mmol) and DMP (24.1 mg, 0.057 mmol) in 0.50 mL DCM for the oxidation step; monomethyl auristatin E (7.4 mg, 0.010 mmol, 1.0 equiv) and $NaBH_3CN$ (2.4 mg, 0.038 mmol, 3.8 equiv) in MeOH (0.30 mL) were used for the reductive amination step. Reverse-phase prep HPLC (100 to 40% H_2O/MeCN, 20 mL/min, 45 min) followed by lyophilization from H_2O/MeCN provided **6** (4.7 mg TFA salt, 34% yield) as a yellow powder.
^{1}H NMR (500 MHz, DMSO-*d*$_6$) (Overlapping mixture of rotamers obscures NMR assignments) δ 11.08 (s, 1H), 8.13 – 8.00 (m, 2H), 7.89 (d, *J* = 8.7 Hz, 1H), 7.64 – 7.55 (m, 2H), 7.36 – 7.23 (m, 8H), 7.20 – 7.11 (m, 3H), 7.04 (d, *J* = 7.0 Hz, 1H), 6.73 (t, *J* = 4.7 Hz, 1H), 6.60 (t, *J* = 5.9 Hz, 1H), 5.41 (d, *J* = 5.1 Hz, 1H), 5.34 (d, *J* = 4.9 Hz, 1H), 5.05 (dd, *J* = 12.7, 5.4 Hz, 1H), 4.79 – 4.72 (m, 1H), 4.67 – 4.40 (m, 5H), 4.07 – 3.89 (m, 4H), 3.78 (dd, *J* = 9.3, 2.4 Hz, 1H), 3.62 (t, *J* = 5.4 Hz, 3H), 3.58 – 3.40 (m, 40H), 3.25 (s, 4H), 3.23 (s, 3H), 3.20 (s, 4H), 3.17 (s, 3H), 3.14 (s, 3H), 3.08 – 3.02 (m, 3H), 2.98 (br s, 2H), 2.88 (ddd, *J* = 16.7, 13.8, 5.3 Hz, 1H), 2.76 – 2.64 (m, 4H), 2.62 – 2.55 (m, 1H), 2.54 (s, 5H), 2.41 (d, *J* = 16.0 Hz, 2H), 2.28 – 2.20 (m, 7H), 2.13 (ddd, *J* = 9.8, 6.9, 4.3 Hz, 2H), 2.06 – 1.85 (m, 6H), 1.85 – 1.68 (m, 6H), 1.62 – 1.45 (m, 4H), 1.17 (t, *J* = 7.2 Hz, 1H), 1.08 – 0.95 (m, 12H), 0.95 – 0.80 (m, 28H), 0.79 – 0.63 (m, 12H).
MS (ESI) *m/z*: 1236.80 $(M+H)^+$.

1-amino-3,6,9,12,15,18,21,24-octaoxaheptacosan-27-ol (**S8**)

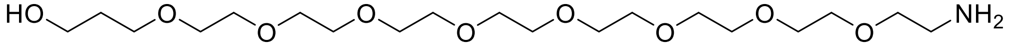

A solution of amino-PEG8-*t*-butyl ester (32.5 mg, 0.065 mmol, 1.0 equiv) in anhydrous THF (0.5 mL) was sparged with N_2 for 5 minutes and then cooled to 0 °C. Lithium aluminum hydride (2 M solution in THF, 0.060 mL, 0.120 mmol, 1.85 equiv) was added dropwise over 3 minutes. The reaction was stirred for 2 hours, warming slowly to room temperature. The reaction was quenched with wet ethyl acetate. $MgSO_4$ was added, and the mixture was filtered, washing with methanol. The filtrate was concentrated to provide **S8** (16.2 mg, 58% yield) as a colorless oil which was used in the next step without purification.
2-(2,6-dioxopiperidin-3-yl)-4-((27-hydroxy-3,6,9,12,15,18,21,24-octaoxaheptacosyl)amino)isoindoline-1,3-dione (**S9**)

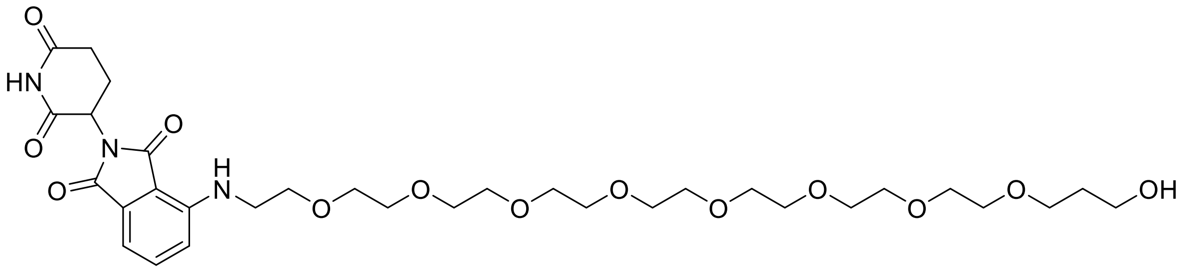

The title compound was prepared according to General Procedure A using **S1** (28.1 mg, 0.10 mmol, 2.5 equiv), **S8** (16.2 mg, 0.04 mmol, 1.0 equiv), and DIPEA (15.00 µL, 0.09 mmol, 2.25 equiv) in DMSO (0.6 mL) at 120 °C for 4 hours. ISCO flash chromatography (4 g silica, 0 – 10% MeOH/DCM, 6 minute gradient then 5 minute isocratic hold) provided **S9** (9.9 mg, 38% yield) as a yellow oil.
^{1}H NMR (500 MHz, $CDCl_3$) δ 8.39 (s, 1H), 7.49 (dd, *J* = 8.5, 7.1 Hz, 1H), 7.10 (d, *J* = 7.1 Hz, 1H), 6.92 (d, *J* = 8.5 Hz, 1H), 6.50 (t, *J* = 5.6 Hz, 1H), 4.90 (dd, *J* = 12.2, 5.2 Hz, 1H), 3.76 (t, *J* = 5.6 Hz, 2H), 3.72 (t, *J* = 5.5 Hz,

2H), 3.70 – 3.58 (m, 30H), 3.46 (q, J = 5.5 Hz, 2H), 2.92 – 2.85 (m, 1H), 2.84 – 2.71 (m, 2H), 2.61 (s, 1H), 2.15 – 2.09 (m, 1H), 1.82 (p, J = 5.6 Hz, 2H).
MS (ESI) *m/z*: 684.50 $(M+H)^+$.

(29S)-1-((2-(2,6-dioxopiperidin-3-yl)-1,3-dioxoisoindolin-4-yl)amino)-N-((S)-1-(((3R,4S,5S)-1-((S)-2-((1R,2R)-3-(((1S,2R)-1-hydroxy-1-phenylpropan-2-yl)amino)-1-methoxy-2-methyl-3-oxopropyl)pyrrolidin-1-yl)-3-methoxy-5-methyl-1-oxoheptan-4-yl)(methyl)amino)-3-methyl-1-oxobutan-2-yl)-29-isopropyl-28-methyl-3,6,9,12,15,18,21,24-octaoxa-28-azatriacontan-30-amide (**7, BJG-03-089**)

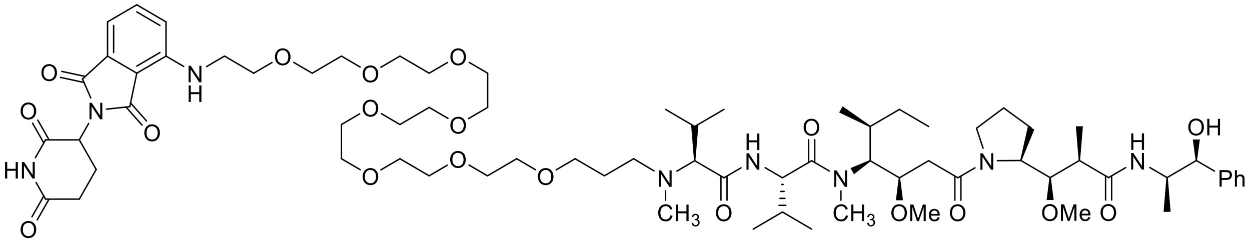

The title compound was prepared according to General Procedure C using **S9** (9.9 mg, 0.015 mmol) and DMP (13.1 mg, 0.031 mmol) in 0.30 mL DCM for the oxidation step; monomethyl auristatin E (7.1 mg, 0.010 mmol, 1.0 equiv) and $NaBH_3CN$ (3.8 mg, 0.060 mmol, 6.0 equiv) in MeOH (0.30 mL) were used for the reductive amination step. Reverse-phase prep HPLC (100 to 40% H_2O/MeCN, 20 mL/min, 45 min) followed by lyophilization from H_2O/MeCN provided **7** (1.0 mg TFA salt, 7% yield) as a yellow powder.
MS (ESI) *m/z*: 692.60 $([M+2]/2)^+$ (Exact mass = 1382.82).

10-((2-(2,6-dioxopiperidin-3-yl)-1,3-dioxoisoindolin-4-yl)amino)decanoic acid (**S10**)

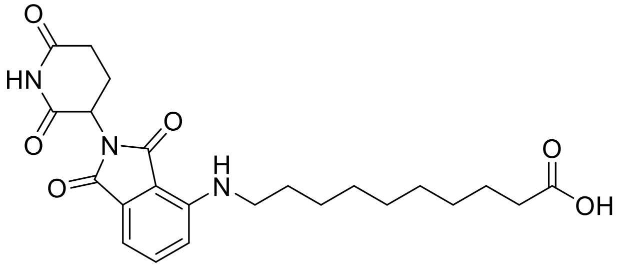

The title compound was prepared according to General Procedure A using **S1** (27.4 mg, 0.10 mmol, 1.0 equiv), 10-aminodecanoic acid TFA salt (41.7 mg, 0.13 mmol, 1.3 equiv), and DIPEA (70.0 μL, 0.40 mmol, 4.0 equiv) in DMSO (0.60 mL) at 120 °C overnight. ISCO flash chromatography (4g, 0–15% MeOH/DCM gradient, 12 min) provided **S10** (34.7 mg, 78% yield) as a yellow film.
^{1}H NMR (500 MHz, DMSO-d_6, two protons hidden under water peak) δ 11.93 (s, 1H), 11.08 (s, 1H), 7.58 (dd, J = 8.6, 7.0 Hz, 1H), 7.09 (d, J = 8.6 Hz, 1H), 7.01 (d, J = 7.0 Hz, 1H), 6.52 (t, J = 5.9 Hz, 1H), 5.05 (dd, J = 12.7, 5.4 Hz, 1H), 2.88 (ddt, J = 16.9, 13.7, 5.5 Hz, 1H), 2.63 – 2.53 (m, 2H), 2.17 (t, J = 7.4 Hz, 2H), 2.06 – 1.98 (m, 1H), 1.57 (p, J = 7.0 Hz, 2H), 1.51 – 1.43 (m, 2H), 1.37 – 1.19 (m, 10H).
MS (ESI) *m/z*: 444.28 $(M+H)^+$.

10-((2-(2,6-dioxopiperidin-3-yl)-1,3-dioxoisoindolin-4-yl)amino)-N-((S)-1-(((S)-1-(((3R,4S,5S)-1-((S)-2-((1R,2R)-3-(((1S,2R)-1-hydroxy-1-phenylpropan-2-yl)amino)-1-methoxy-2-methyl-3-oxopropyl)pyrrolidin-1-yl)-3-methoxy-5-methyl-1-oxoheptan-4-yl)(methyl)amino)-3-methyl-1-oxobutan-2-yl)amino)-3-methyl-1-oxobutan-2-yl)-N-methyldecanamide (**8, BJG-03-097**)

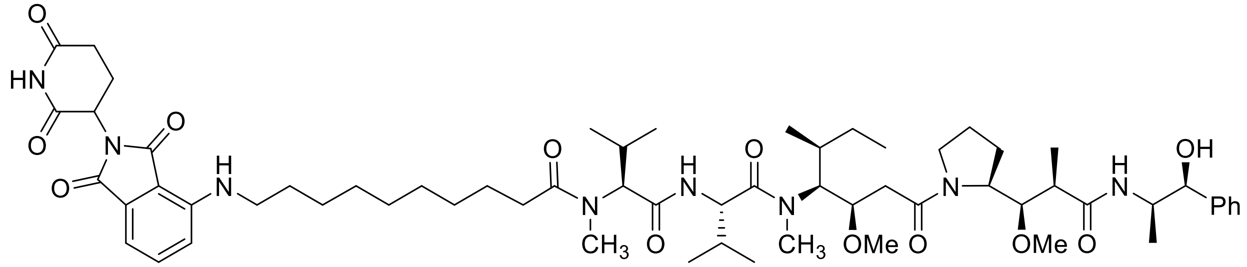

The title compound was prepared according to General Procedure B using **S10** (7.1 mg, 0.015 mmol, 1.5 equiv), monomethyl auristatin E (7.2 mg, 0.010 mmol, 1.0 equiv), HATU (7.0 mg, 0.018 mmol, 1.8 equiv) and DIPEA (6.00 μL, 0.034 mmol, 3.4 equiv) in 0.30 mL DMSO at room temperature overnight. Reverse-phase prep HPLC (80 to 0% H_2O/MeCN, 20 mL/min, 45 min) followed by lyophilization from H_2O/MeCN provided **8** (5.0 mg, TFA salt, 39% yield) as a fluffy yellow powder.

^{1}H NMR (500 MHz, DMSO-d_6) δ 11.08 (s, 1H), 8.54 (rotamer, t, *J* = 10.6 Hz, 0.5H), 7.89 (rotamer, dd, *J* = 8.8, 4.6 Hz, 0.5H), 7.73 – 7.52 (m, 2H), 7.34 – 7.28 (m, 2H), 7.28 – 7.21 (m, 4H), 7.20 – 7.12 (m, 3H), 7.07 (d, *J* = 15.1 Hz, 3H), 7.01 (d, *J* = 7.1 Hz, 1H), 6.52 (t, *J* = 6.0 Hz, 1H), 5.46 – 5.28 (rotamers, 2 broad singlets, 1H), 5.04 (dd, *J* = 12.7, 5.4 Hz, 1H), 4.67 – 4.34 (m, 2H), 4.07 – 3.89 (m, 2H), 3.63 – 3.53 (m, 1H), 3.32 – 3.08 (m, 12H), 3.00 – 2.79 (m, 5H), 2.58 (d, *J* = 19.0 Hz, 1H), 2.35 – 2.21 (m, 2H), 2.16 – 2.06 (m, 2H), 2.06 – 1.97 (m, 2H), 1.86 – 1.66 (m, 3H), 1.63 – 1.40 (m, 7H), 1.38 – 1.19 (m, 14H), 1.07 – 0.96 (m, 6H), 0.91 – 0.65 (m, 18H).

MS (ESI) *m/z*: 1144.76 $(M+H)^+$.

(E)-3-(3-hydroxy-4-methoxyphenyl)-2-(3,4,5-trimethoxyphenyl)acrylic acid (**S11**)

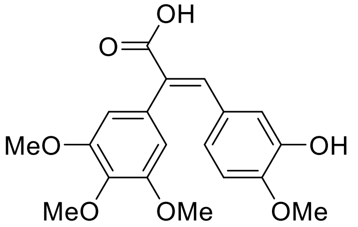

The compound was prepared according to the reported procedure [3]. A solution of 3,4,5-trimethoxyphenylacetic acid (2.715 g, 12.0 mmol, 2 equiv) and 3-hydroxy-4-methoxybenzaldehyde (913 mg, 6.0 mmol, 1 equiv) in triethylamine (3.0 mL) and acetic anhydride (6.0 mL) was heated to 140 °C for 3 hours. The reaction was then cooled, and concentrated HCl (5 mL) was added slowly. The mixture was diluted with 200 mL DCM and extracted with saturated sodium bicarbonate (3x50 mL). The combined aqueous layers were acidified, and the resulting precipitate was collected by suction filtration to provide **S11** (1.223 g, 56% yield) as an off-white solid.

^{1}H NMR (500 MHz, DMSO-d_6) δ 12.41 (s, 1H), 8.94 (s, 1H), 7.56 (s, 1H), 6.80 (d, *J* = 8.5 Hz, 1H), 6.60 (dd, *J* = 8.4, 2.2 Hz, 1H), 6.53 (d, *J* = 2.2 Hz, 1H), 6.43 (s, 2H), 3.73 (s, 3H), 3.71 (s, 3H), 3.68 (s, 6H). MS (ESI) *m/z*: 361.17 $(M+H)^+$.

(Z)-2-methoxy-5-(3,4,5-trimethoxystyryl)phenol (**Combretastatin A-4**, **CA4**)

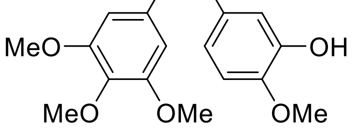

The compound was prepared according to the reported procedure [3]. A suspension of **S11** (400 mg, 1.1 mmol, 1.0 equiv) and copper powder (325 mesh; 609 mg, 9.6 mmol, 8.7 equiv) in quinoline (3.5 mL) was heated to 200 °C for 1 hour. UPLC-MS analysis showed complete decarboxylation. The reaction mixture was diluted with EtOAc (50 mL) and washed with 1 M HCl (4x20 mL), then dried over $MgSO_4$, filtered, and concentrated. ISCO flash chromatography (24 g, 0–100% EtOAc/hexanes, 15 minutes) provided **CA4** (194 mg, 55% yield) as a clear, viscous yellow oil. ^{1}H-NMR analysis showed an approx. 10:1 Z/E alkene ratio.

^{1}H NMR (500 MHz, $CDCl_3$) **Z-isomer**: δ 6.92 (d, *J* = 2.1 Hz, 1H), 6.80 (dd, *J* = 8.4, 2.0 Hz, 1H), 6.73 (d, *J* = 8.3 Hz, 1H), 6.53 (s, 2H), 6.47 (d, *J* = 12.2 Hz, 1H), 6.41 (d, *J* = 12.2 Hz, 1H), 5.49 (s, 1H), 3.87 (s, 3H), 3.84 (s, 3H), 3.70 (s, 6H).

MS (ESI) *m/z*: 317.17 $(M+H)^+$.

tert-Butyl (Z)-(2-(2-(2-(2-methoxy-5-(3,4,5-trimethoxystyryl)phenoxy)ethoxy)ethoxy)ethyl) carbamate (**S12**)

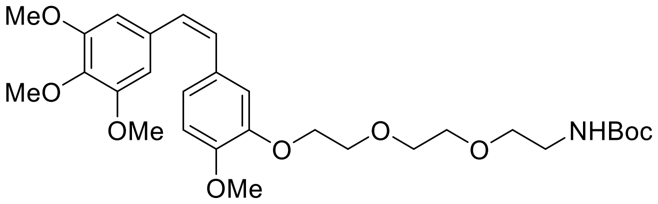

The title compound was prepared according to General Procedure D using CA4 (31.6 mg, 0.10 mmol, 1.0 equiv), triphenylphosphine (27.3 mg, 0.10 mmol, 1.0 equiv), N-Boc-PEG3-alcohol (25.6 mg, 0.10 mmol, 1.0 equiv), and DIAD (20.00 µL, 0.10 mmol, 1.0 equiv) in DMF (0.5 mL) at room temperature for 23 hours. ISCO flash chromatography (4 g, 0–10% MeOH/DCM, 7 minute gradient then 2 minute isocratic hold) provided **S12** (35.9 mg, 65% yield) as a clear, pale yellow oil which partially solidified on standing.

UPLC-MS analysis showed a 17:1 mix of alkene isomers; 1H-NMR analysis confirmed the Z-isomer as the major component.

^{1}H NMR (500 MHz, $CDCl_3$) δ 6.90 – 6.83 (m, 2H), 6.76 (d, *J* = 8.2 Hz, 1H), 6.51 (s, 2H), 6.49 (d, *J* = 12.2 Hz, 1H), 6.44 (d, *J* = 12.1 Hz, 1H), 3.99 (dd, *J* = 5.8, 4.4 Hz, 2H), 3.84 – 3.82 (overlapping singlets, 6H), 3.79 (dd, *J* = 5.8, 4.4 Hz, 2H), 3.70 (s, 6H), 3.68 – 3.64 (m, 2H), 3.60 (dd, *J* = 5.9, 3.4 Hz, 2H), 3.53 (t, *J* = 5.2 Hz, 2H), 3.30 (d, *J* = 5.2 Hz, 2H), 1.43 (s, 9H).

MS (ESI) *m/z*: 570.29 $(M+Na)^+$; 448.28 $(M\text{-}Boc+H)^+$.

(Z)-2-(2,6-dioxopiperidin-3-yl)-4-((2-(2-(2-(2-methoxy-5-(3,4,5-trimethoxystyryl)phenoxy)ethoxy)ethoxy)ethyl)amino)isoindoline-1,3-dione (**9, BJG-03-081**)

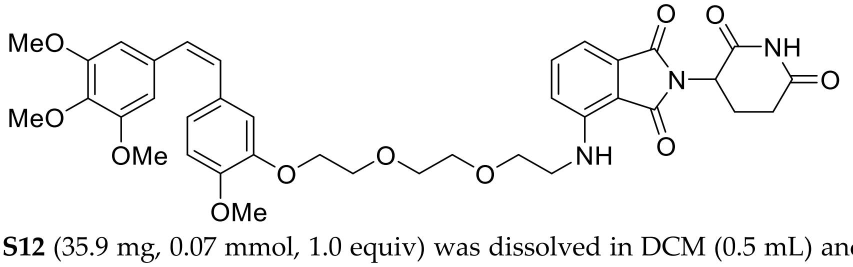

S12 (35.9 mg, 0.07 mmol, 1.0 equiv) was dissolved in DCM (0.5 mL) and TFA (0.1 mL). The reaction was stirred at room temperature for 1 hour, at which time UPLC-MS analysis showed full deprotection. Solvents were removed *in vacuo*. The crude deprotection product was then reacted with **S1** (28.4 mg, 0.10 mmol, 1.4 equiv) and DIPEA (87.0 µL, 0.50 mmol, 7.1 equiv) in DMSO (0.8 mL) at 50 °C for 36 hours, following General

Procedure A. Reverse-phase HPLC (20 mL/min, 100-0% H_2O/MeCN, 45 minute gradient) followed by lyophilization from H_2O/MeCN provided **9** (4.2 mg TFA salt, 7% yield) as a dark yellow powder.

^{1}H NMR (500 MHz, DMSO-d_6) δ 11.02 (s, 1H), 8.33 (s, 1H), 7.55 – 7.44 (m, 1H), 7.17 (s, 1H), 7.07 (s, 2H), 6.99 – 6.93 (m, 3H), 6.83 – 6.73 (m, 3H), 6.53 (t, *J* = 5.9 Hz, 1H), 6.48 (s, 2H), 6.42 (d, *J* = 12.2 Hz, 1H), 6.38 (d, *J* = 12.2 Hz, 1H), 4.97 (dd, *J* = 12.7, 5.4 Hz, 1H), 3.78 (dd, *J* = 5.7, 3.7 Hz, 2H), 3.65 (s, 3H), 3.59 – 3.52 (m, 18H), 3.49 (s, 3H), 3.11 – 3.03 (m, 3H), 2.80 (ddd, *J* = 16.6, 13.7, 5.4 Hz, 1H), 1.97 – 1.89 (m, 1H).

MS (ESI) *m/z*: 704.40 (M+H)$^+$.

(Z)-2-(2-(2-(2-(2-methoxy-5-(3,4,5-trimethoxystyryl)phenoxy)ethoxy)ethoxy)ethoxy)ethan-1-amine trifluoroacetate salt (**S13**)

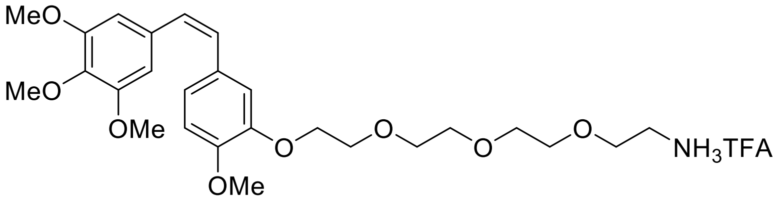

The title compound was prepared according to General Procedure D using N-Boc-PEG4-alcohol (19.5 mg, 0.066 mmol, 1.1 equiv). Modification: added 0.05 mmol DIAD (10.00 μL, 0.9 equiv) after 20 hours to promote full conversion. The crude Mitsunobu product was dissolved in DCM (0.4 mL) and TFA (0.1 mL). The reaction was stirred at room temperature overnight. Solvents were removed *in vacuo*. Preparative reverse-phase HPLC (20 mL/min, 80-35% H_2O/MeCN, 28 minute gradient) provided **S13** (7.4 mg TFA salt, 20% yield, 2 steps) as a yellow oil.

^{1}H NMR (500 MHz, DMSO-d_6) δ 6.89 (d, *J* = 8.3 Hz, 1H), 6.87 – 6.84 (m, 1H), 6.82 (d, *J* = 1.9 Hz, 1H), 6.55 (s, 2H), 6.50 (d, *J* = 12.2 Hz, 1H), 6.45 (d, *J* = 12.2 Hz, 1H), 3.85 (dd, *J* = 5.7, 3.7 Hz, 2H), 3.74 (s, 3H), 3.64 (s, 3H), 3.61 (s, 6H), 3.60 – 3.51 (m, 12H), 2.97 (h, *J* = 5.7 Hz, 2H).

MS (ESI) *m/z*: 492.28 (M+H)$^+$.

(Z)-2-(2,6-dioxopiperidin-3-yl)-4-((2-(2-(2-(2-(2-methoxy-5-(3,4,5-trimethoxystyryl)phenoxy)ethoxy)ethoxy)ethoxy)ethyl)amino)isoindoline-1,3-dione (**10, BJG-03-033-1**)

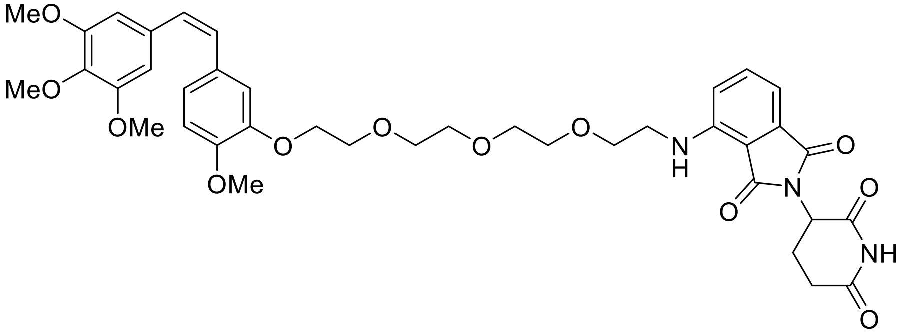

The title compound was prepared according to General Procedure A using **S13** (7.4 mg, 0.0122 mmol, 1.0 equiv), **S1** (6.8 mg, 0.024 mmol, 2.0 equiv), and DIPEA (12.00 μL, 0.069 mmol, 5.6 equiv) in DMSO (0.4 mL) at 80 °C for 24 hours. Preparative reverse-phase HPLC (20 mL/min, 100-0% H_2O/MeCN, 45 minute gradient) followed by lyophilization from H_2O/MeCN provided **10** (1.9 mg TFA salt, 18% yield) as a yellow solid.

^{1}H NMR (500 MHz, DMSO-d_6) δ 11.08 (s, 1H), 7.61 – 7.52 (m, 1H), 7.20 (s, 1H), 7.15 – 7.06 (m, 2H), 7.03 (d, *J* = 7.1 Hz, 1H), 6.99 (s, 1H), 6.89 (d, *J* = 8.3 Hz, 1H), 6.84 (dd, *J* = 8.3, 1.9 Hz, 1H), 6.82 (d, *J* = 1.9 Hz, 1H), 6.60

(t, *J* = 5.8 Hz, 1H), 6.55 (s, 2H), 6.50 (d, *J* = 12.2 Hz, 1H), 6.45 (d, *J* = 12.2 Hz, 1H), 5.05 (dd, *J* = 12.7, 5.4 Hz, 1H), 3.84 (dd, *J* = 5.7, 3.8 Hz, 2H), 3.72 (s, 3H), 3.63 (s, 3H), 3.63 – 3.61 (m, 2H), 3.60 (s, 6H), 3.57 – 3.49 (m, 9H), 3.45 (q, *J* = 5.6 Hz, 2H), 2.92 – 2.83 (m, 1H), 2.67 – 2.51 (m, 2H), 2.05 – 1.95 (m, 1H).
MS (ESI) *m/z*: 748.41 $(M+H)^+$.

(Z)-14-(2-methoxy-5-(3,4,5-trimethoxystyryl)phenoxy)-3,6,9,12-tetraoxatetradecan-1-amine trifluoroacetate salt (**S14**)

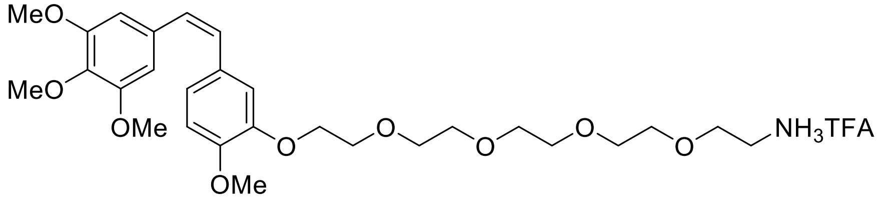

The title compound was prepared according to General Procedure D using **CA4** (54.5 mg, 0.16 mmol, 1.0 equiv), triphenylphosphine (43.1 mg, 0.16 mmol, 1.0 equiv), and N-Boc-PEG5-alcohol (59.8 mg, 0.16 mmol, 1.0 equiv) in DMF (0.35 mL), and DIAD (32.0 μL, 0.16 mmol, 1.0 equiv) at room temperature overnight. The crude Mitsunobu product, a waxy white solid, was dissolved in DCM (0.5 mL) and TFA (0.12 mL). The reaction was stirred at room temperature for 1 hour, at which time UPLC-MS analysis showed full deprotection. Solvents were removed *in vacuo*. Preparative reverse-phase HPLC (20 mL/min, 80-35% H_2O/MeCN, 28 minute gradient) provided **S14** (30.7 mg TFA salt, 30% yield, 2 steps) a gold oil.
Note: All attempts to prepare the degraders by direct alkylation of CA4 with either hydroxy-PEG(n)-thalidomide or iodo-PEG(n)-thalidomide were unsuccessful.
^{1}H-NMR (**Boc-protected intermediate**) (500 MHz, $CDCl_3$) δ 6.87 (dd, *J* = 8.2, 2.0 Hz, 1H), 6.85 (d, *J* = 2.0 Hz, 1H), 6.76 (d, *J* = 8.2 Hz, 1H), 6.51 (s, 2H), 6.49 (d, *J* = 12.1 Hz, 1H), 6.43 (d, *J* = 12.1 Hz, 1H), 3.99 (t, *J* = 5.2 Hz, 2H), 3.83 (s, 6H), 3.81 – 3.77 (m, 2H), 3.70 (s, 6H), 3.68 – 3.58 (m, 12H), 3.53 (t, *J* = 5.3 Hz, 2H), 3.34 – 3.27 (m, 2H), 1.44 (s, 9H).
MS (ESI, **Boc-protected intermediate**) *m/z*: 657.90 $(M+Na)^+$.
MS (ESI, **Deprotection**) *m/z*: 536.64 $(M+H)^+$.

(Z)-2-(2,6-dioxopiperidin-3-yl)-4-((14-(2-methoxy-5-(3,4,5-trimethoxystyryl)phenoxy)-3,6,9,12-tetraoxatetradecyl)amino)isoindoline-1,3-dione **11, BJG-02-158**)

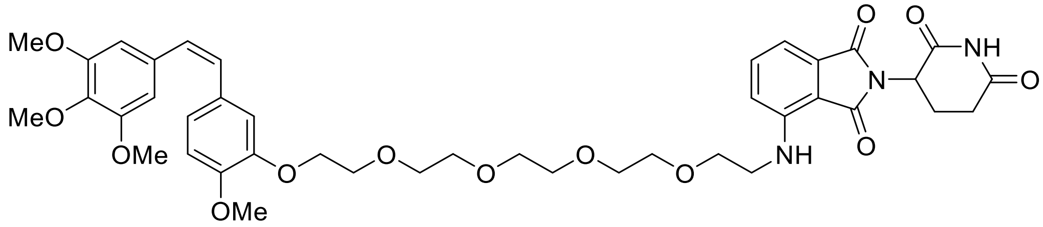

The title compound was prepared according to General Procedure A using **S14** (12.8 mg, 0.02 mmol, 1.0 equiv), **S1** (6.8 mg, 0.025 mmol, 1.2 equiv), and DIPEA (16.00 μL, 0.09 mmol, 4.5 equiv) in DMSO (0.3 mL) at 80 °C for 21 hours. Reverse-phase HPLC (20 mL/min, 100 – 0% H_2O/MeOH, 45 minute gradient) followed by preparative TLC (5% MeOH/DCM) and lyophilization from H_2O/MeCN provided **11** (3.0 mg TFA salt, 17% yield) as a yellow powder.
^{1}H NMR (Z-isomer only) (500 MHz, DMSO-*d*$_6$) δ 11.08 (s, 1H), 7.62 – 7.49 (m, 1H), 7.15 – 7.05 (m, 2H), 7.03 (dd, *J* = 7.0, 2.3 Hz, 1H), 6.90 – 6.79 (m, 3H), 6.59 (t, *J* = 5.2 Hz, 1H), 6.55 (s, 2H), 6.50 (d, *J* = 12.2 Hz, 1H), 6.45 (d, *J* = 12.2 Hz, 1H), 5.05 (dd, *J* = 12.6, 5.3 Hz, 1H), 4.13 (t, *J* = 4.8 Hz, 1H), 3.84 (dd, *J* = 5.7, 3.8 Hz, 2H), 3.82

(s, 3H), 3.72 (s, 3H), 3.62 – 3.57 (m, 10H), 3.56 – 3.43 (m, 14H), 2.93 – 2.82 (m, 1H), 2.65 – 2.52 (m, 2H), 2.05 – 1.95 (m, 1H).
MS (ESI) *m/z*: 791.71 $(M+H)^+$.

(Z)-17-(2-methoxy-5-(3,4,5-trimethoxystyryl)phenoxy)-3,6,9,12,15-pentaoxaheptadecan-1-amine trifluoroacetate salt (**S15**)

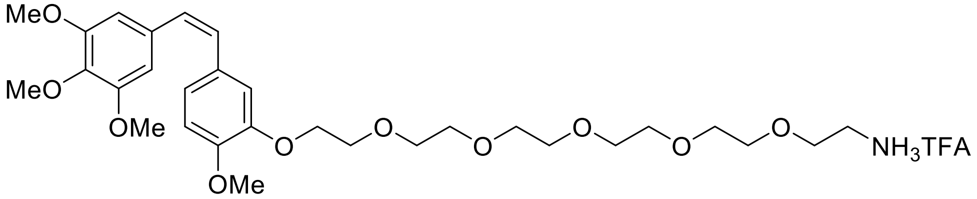

The title compound was prepared according to General Procedure D using N-Boc-PEG6-alcohol (25.6 mg, 0.066 mmol, 1.1 equiv). Modification: added 0.05 mmol DIAD (10.00 µL, 0.9 equiv) after 20 hours to promote full conversion. The crude Mitsunobu product was dissolved in DCM (0.4 mL) and TFA (0.1 mL). The reaction was stirred at room temperature overnight. Solvents were removed *in vacuo*. Preparative reverse-phase HPLC (20 mL/min, 80-35% H_2O/MeCN, 28 minute gradient) provided **S15** (6.6 mg TFA salt, 16% yield, 2 steps) as a yellow oil.
^1H NMR (500 MHz, DMSO-d_6) δ 6.89 (d, *J* = 8.3 Hz, 1H), 6.87 – 6.84 (m, 1H), 6.82 (d, *J* = 1.9 Hz, 1H), 6.54 (s, 2H), 6.50 (d, *J* = 12.2 Hz, 1H), 6.45 (d, *J* = 12.2 Hz, 1H), 3.85 (dd, *J* = 5.7, 3.7 Hz, 2H), 3.74 (s, 3H), 3.64 (s, 3H), 3.61 (s, 6H), 3.60 – 3.48 (m, 20H), 2.97 (q, *J* = 5.5 Hz, 2H).
MS (ESI) *m/z*: 580.39 $(M+H)^+$.

(Z)-2-(2,6-dioxopiperidin-3-yl)-4-((17-(2-methoxy-5-(3,4,5-trimethoxystyryl)phenoxy)-3,6,9,12,15-pentaoxaheptadecyl)amino)isoindoline-1,3-dione (**12, BJG-03-033-2**)

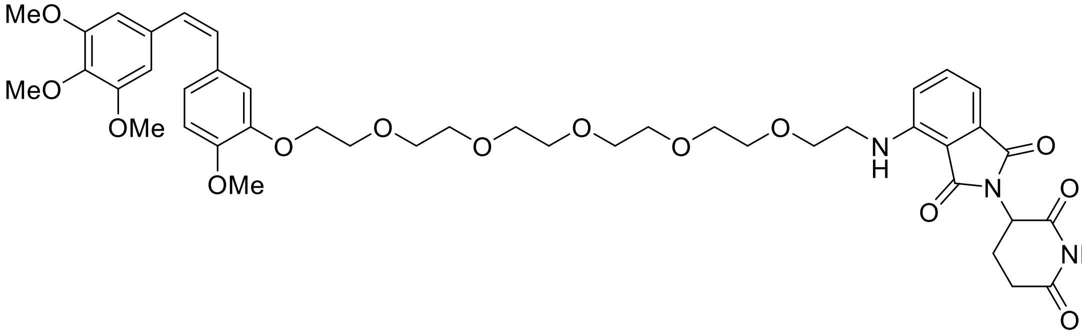

The title compound was prepared according to General Procedure A using **S15** (6.6 mg, 0.0095 mmol, 1.0 equiv), **S1** (7.3 mg, 0.0264 mmol, 2.7 equiv), and DIPEA (12.00 µL, 0.069 mmol, 7.2 equiv) in DMSO (0.4 mL) at 80 °C for 24 hours. Preparative reverse-phase HPLC (20 mL/min, 100-0% H_2O/MeCN, 45 minute gradient) followed by lyophilization from H_2O/MeCN provided **12** (4.4 mg TFA salt, 48% yield) as a yellow solid.
^1H NMR (500 MHz, DMSO-d_6) δ 11.08 (s, 1H), 7.57 (t, *J* = 8.6, 7.0 Hz, 1H), 7.22 (s, 1H), 7.15 – 7.10 (m, 2H), 7.06 – 6.99 (m, 2H), 6.91 – 6.80 (m, 2H), 6.60 (t, *J* = 5.7 Hz, 1H), 6.55 (d, *J* = 4.7 Hz, 2H), 6.50 (d, *J* = 12.2 Hz, 1H), 6.45 (d, *J* = 12.1 Hz, 1H), 5.05 (dd, *J* = 12.7, 5.4 Hz, 1H), 3.85 (dd, *J* = 5.7, 3.8 Hz, 2H), 3.73 (s, 3H), 3.63 (s, 3H), 3.63 – 3.58 (m, 8H), 3.58 – 3.43 (m, 20H), 2.88 (ddd, *J* = 16.8, 13.6, 5.4 Hz, 1H), 2.65 – 2.51 (m, 2H), 2.02 (ddd, *J* = 13.4, 5.9, 3.5 Hz, 1H).
MS (ESI) *m/z*: 836.41 $(M+H)^+$.

(E)-3-(4-methoxy-3-nitrophenyl)-2-(3,4,5-trimethoxyphenyl)acrylic acid (**S16**)

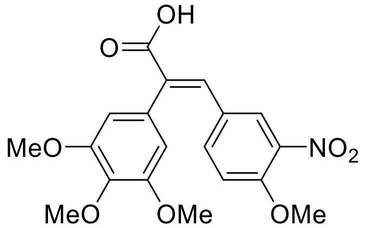

The compound was prepared in the same manner as **S11**. A solution of 3,4,5-trimethoxyphenylacetic acid (2.712 g, 12.0 mmol, 2.0 equiv) and 4-methoxy-3-nitrobenzaldehyde (1.089 g, 6.0 mmol, 1.0 equiv) in triethylamine (3.0 mL) and acetic anhydride (6.0 mL) was heated to 140 °C for 2.5 hours. After cooling to room temperature, the reaction was acidified with concentrated HCl (6 mL) and left overnight. The next morning, the resulting precipitate was collected by suction filtration, washing with H_2O/MeOH (3:1, 100 mL) and dried *in vacuo* to provide **S16** (1.863 g, 79% yield) as a light brown powder.

^{1}H NMR (500 MHz, DMSO-d_6) δ 12.65 (s, 1H), 7.70 (s, 1H), 7.53 (d, *J* = 2.3 Hz, 1H), 7.41 (dd, *J* = 9.0, 2.1 Hz, 1H), 7.25 (d, *J* = 8.9 Hz, 1H), 6.47 (s, 2H), 3.89 (s, 3H), 3.71 (s, 3H), 3.69 (s, 6H).

MS (ESI) *m*/*z*: 390.27 $(M+H)^+$.

(Z)-1,2,3-trimethoxy-5-(4-methoxy-3-nitrostyryl)benzene (**S17**)

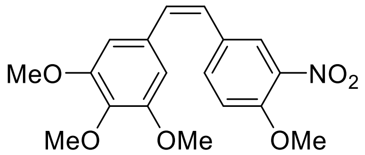

The compound was prepared in the same manner as **CA4**. A suspension of **S16** (784 mg, 2.0 mmol, 1.0 equiv) and copper powder (1.109 g, 17.5 mmol, 8.75 equiv, 325 mesh) in quinoline (6.5 mL) was heated to 200 °C for 1.75 hours. The reaction mixture was diluted with EtOAc (50 mL) and washed with 1 M HCl (4x20 mL), then dried over $MgSO_4$, filtered, and concentrated. ISCO flash chromatography (40 g, 0–100% EtOAc/hexanes, 18 minutes) provided **S17** (372 mg, 43% yield) as a dark red, viscous oil. ^{1}H-NMR analysis matched the reported spectrum [4] and showed a 4.5:1 Z/E alkene ratio.

^{1}H NMR (500 MHz, DMSO-d_6) δ 7.77 (d, *J* = 2.2 Hz, 1H), 7.54 (dd, *J* = 8.7, 2.3 Hz, 1H), 7.29 (d, *J* = 8.8 Hz, 1H), 6.60 (d, *J* = 12.2 Hz, 1H), 6.55 (d, *J* = 12.0 Hz, 1H), 6.54 (s, 2H), 3.90 (s, 3H), 3.66 (s, 3H), 3.62 (s, 6H).

MS (ESI) *m*/*z*: 346.27 $(M+H)^+$.

(Z)-2-methoxy-5-(3,4,5-trimethoxystyryl)aniline (**S18**)

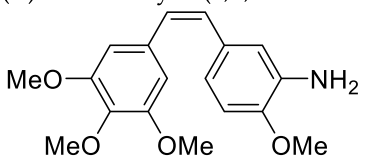

The compound was prepared following a reported procedure [4]. A suspension of **S17** (155.7 mg, 0.45 mmol, 1.0 equiv) and nickel(II) acetate tetrahydrate (228 mg, 0.91 mmol, 2.0 equiv) in methanol (3.5 mL) was cooled on ice. To the stirred solution, $NaBH_4$ was added in four portions at 15-minute intervals (20.9 mg, 12.5 mg, 14.3 mg, 7.2 mg; total 54.9 mg, 1.45 mmol, 3.2 equiv), maintaining the temperature at 0 °C throughout. After the final $NaBH_4$ addition, the reaction was stirred for 5 minutes and then diluted with methanol. The crude material was dry-loaded directly onto silica gel and purified by ISCO flash

chromatography (12 g, 0–100% EtOAc/hexanes, 15 minutes) to provide **S18** (96.1 mg, 67% yield) as a clear amber oil. ^{1}H-NMR analysis matched the reported spectrum.
^{1}H NMR (500 MHz, $CDCl_3$) δ 6.70 (d, *J* = 1.3 Hz, 1H), 6.69 – 6.67 (m, 2H), 6.55 (s, 2H), 6.47 (d, *J* = 12.6 Hz, 1H), 6.37 (d, *J* = 12.7 Hz, 1H), 3.84 (s, 3H), 3.83 (s, 3H), 3.70 (s, 6H).
MS (ESI) *m/z*: 316.17 $(M+H)^+$.

(Z)-N1-(2-methoxy-5-(3,4,5-trimethoxystyryl)phenyl)-3,6,9,12-tetraoxatetradecane-1,14-diamine trifluoroacetate salt (**S19**)

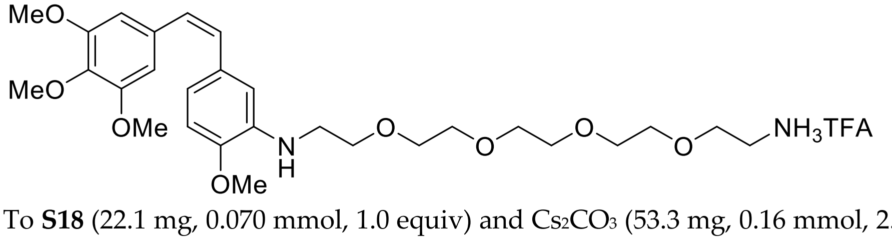

To **S18** (22.1 mg, 0.070 mmol, 1.0 equiv) and Cs_2CO_3 (53.3 mg, 0.16 mmol, 2.3 equiv) was added a solution of N-Boc-PEG4-iodide (43.8 mg, 0.098 mmol, 1.4 equiv) in DMF (0.6 mL). The mixture was heated to 50 °C overnight. UPLC-MS analysis showed incomplete conversion; Cs_2CO_3 (39 mg, 0.12 mmol, 1.7 equiv) were added, and the reaction was heated to 80 °C for 2.5 hours. The reaction was then cooled, and diluted with DCM and H_2O (10 mL each). The aqueous component was extracted with DCM (2x5 mL). The combined organic layers were washed twice with water, then with brine, dried over $MgSO_4$, filtered, and concentrated to provide a clear amber oil. The alkylation product, **S18**, and N-Boc-PEG4-iodide were inseparable by silica chromatography. The mixture was dissolved in DCM (0.4 mL) and TFA (0.1 mL) and stirred at room temperature for 1.5 hours. Solvents were removed *in vacuo*. The material was purified by preparative reverse-phase HPLC (20 mL/min, 100-25% H_2O/MeCN, 45 minute gradient) to provide **S19** (12.8 mg TFA salt, 28% yield, 2 steps) as a clear, colorless oil.
MS (ESI) *m/z*: 535.68 $(M+H)^+$.

(Z)-2-(2,6-dioxopiperidin-3-yl)-4-((14-((2-methoxy-5-(3,4,5-trimethoxystyryl)phenyl)amino)-3,6,9,12-tetraoxatetradecyl)amino)isoindoline-1,3-dione (**13, BJG-03-144**)

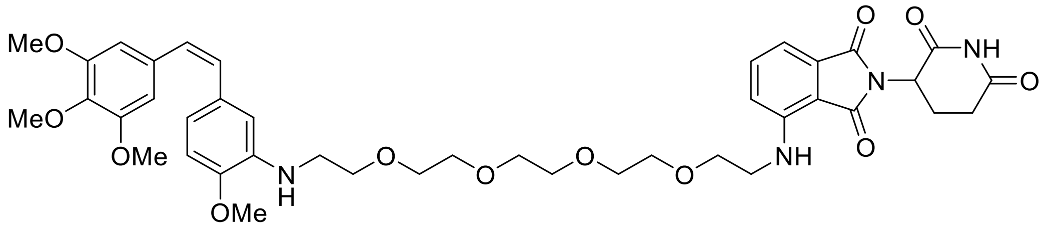

The title compound was prepared according to General Procedure A using **S19** (12.8 mg, 0.020 mmol, 1.0 equiv), **S1** (20.6 mg, 0.075 mmol, 3.75 equiv), DIPEA (40.00 µL, 0.23 mmol, 11.5 equiv) in DMSO (0.8 mL) at 80 °C for 22 hours. Preparative reverse-phase HPLC (20 mL/min, 100-25% H_2O/MeCN, 45 minute gradient) followed by lyophilization from H_2O/MeCN provided **13** (1.2 mg TFA salt, 7% yield) as a yellow solid.
MS (ESI) *m/z*: 791.51 $(M+H)^+$.

2-chloro-5-nitro-N-(2-((3-phenoxybenzyl)amino)pyridin-4-yl)benzamide (**T007-1**)

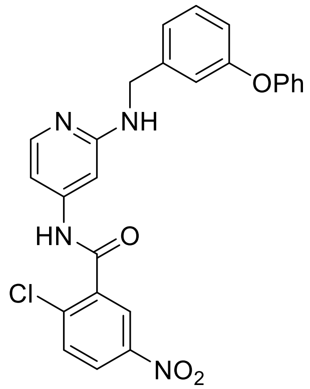

T007-1 was prepared by a modification to the procedure reported by Yang et al [5]. The reported amide coupling with 2-chloro-5-nitrobenzoic acid and DCC led to inseparable mixtures of mono- and diacylation. The final acylation step was achieved using 2-chloro-5-nitrobenzoyl chloride (44.7 mg, 0.20 mmol, 1.0 equiv), N2-(3-phenoxybenzyl)pyridine-2,4-diamine (63.1 mg, 0.22 mmol, 1.1 equiv) and triethylamine (56.0 μL, 0.40 mmol, 2.0 equiv) in DCM (2.0 mL) at 0 °C, warming to room temperature over three hours. ISCO flash chromatography (12 g, 0–10% MeOH/DCM, 15 minutes) provided **T007-1** (38.7 mg, 40% yield) as a yellow solid. The ^{1}H-NMR matched the reported spectrum.

^{1}H NMR (500 MHz, DMSO-*d*$_6$) δ 10.71 (s, 1H), 8.44 (d, *J* = 2.7 Hz, 1H), 8.33 (dd, *J* = 8.8, 2.8 Hz, 1H), 7.90 – 7.84 (m, 2H), 7.39 – 7.33 (m, 2H), 7.30 (t, *J* = 7.8 Hz, 1H), 7.17 (t, *J* = 6.2 Hz, 1H), 7.14 – 7.07 (m, 2H), 7.06 – 7.02 (m, 1H), 6.99 – 6.94 (m, 3H), 6.82 (dd, *J* = 8.2, 2.6 Hz, 1H), 6.70 (dd, *J* = 5.6, 1.8 Hz, 1H), 4.47 (d, *J* = 6.1 Hz, 2H).

MS (ESI) *m*/*z*: 475.28 $(M+H)^+$.

2,3,4,5,6-pentafluoro-N-(3-fluoro-4-methoxyphenyl)benzenesulfonamide (**T138067, batabulin**)

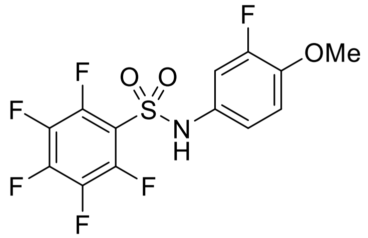

T138067 was prepared following the reported procedure [6] using pentafluorobenzenesulfonyl chloride and 3-fluoro-4-methoxyaniline. To a solution of 3-fluoro-4-methoxyaniline (70.5 mg, 0.50 mmol, 1.0 equiv) in pyridine (1.0 mL) at 0 °C, pentafluorobenzenesulfonyl chloride (0.080 mL, 0.55 mmol, 1.1 equiv) was added dropwise. The reaction was warmed to room temperature over three hours, and then diluted with water (20 mL) and extracted with ethyl acetate (3x10 mL). The combined organic layers were washed with water (twice), 1 M HCl, and brine, then dried over $MgSO_4$, filtered, and concentrated. ISCO flash chromatography (12 g, 0–5% MeOH/DCM, 12 minutes) provided **T138067** (105.7 mg, 57% yield) as an off-white solid.

^{1}H NMR (500 MHz, DMSO-*d*$_6$) δ 11.13 (s, 1H), 7.11 (t, *J* = 9.2 Hz, 1H), 7.01 (dd, *J* = 12.4, 2.6 Hz, 1H), 6.93 (ddd, *J* = 8.9, 2.6, 1.4 Hz, 1H), 3.79 (s, 3H).

^{19}F NMR (471 MHz, DMSO-*d*$_6$) δ -133.00 (t, *J* = 10.9 Hz), -137.24 – -137.77 (m), -145.59 – -146.25 (m), -159.43 (tt, *J* = 19.4, 12.0 Hz).

MS (ESI) *m*/*z*: 370.87 $(M+H)^+$.

5. References

1. Nowak, R.P.; DeAngelo, S.L.; Buckley, D.; He, Z.; Donovan, K.A.; An, J.; Safaee, N.; Jedrychowski, M.P.; Ponthier, C.M.; Ishoey, M., et al. Plasticity in binding confers selectivity in ligand-induced protein degradation. *Nat Chem Biol* **2018**, *14*, 706-714, doi:10.1038/s41589-018-0055-y.
2. Carpenter, A.E.; Jones, T.R.; Lamprecht, M.R.; Clarke, C.; Kang, I.H.; Friman, O.; Guertin, D.A.; Chang, J.H.; Lindquist, R.A.; Moffat, J., et al. CellProfiler: image analysis software for identifying and quantifying cell phenotypes. *Genome Biol* **2006**, *7*, R100, doi:10.1186/gb-2006-7-10-r100.
3. Gaukroger, K.; Hadfield, J.A.; Hepworth, L.A.; Lawrence, N.J.; McGown, A.T. Novel syntheses of cis and trans isomers of combretastatin A-4. *J. Org. Chem.* **2001**, *66*, 8135-8138, doi:10.1021/jo015959z.
4. Pettit, G.R.; Rhodes, M.R.; Herald, D.L.; Hamel, E.; Schmidt, J.M.; Pettit, R.K. Antineoplastic agents. 445. Synthesis and evaluation of structural modifications of (Z)- and (E)-combretastatin A-41. *J. Med. Chem.* **2005**, *48*, 4087-4099, doi:10.1021/jm0205797.
5. Yang, J.; Li, Y.; Yan, W.; Li, W.; Qiu, Q.; Ye, H.; Chen, L. Covalent modification of Cys-239 in beta-tubulin by small molecules as a strategy to promote tubulin heterodimer degradation. *J. Biol. Chem.* **2019**, *294*, 8161-8170, doi:10.1074/jbc.RA118.006325.
6. Tularik Inc.; Flygare, J.A.M., Julio C.; Shan, Bei; Clark, David L.; Rosen, Terry J. . Pentafluorobenzenesulfonamides and analogs. *US Patent US6482860 B1* **2002**.
